# Regional changes in psychotropic use among Finnish persons with newly diagnosed Alzheimer’s disease in 2005-2011

**DOI:** 10.1371/journal.pone.0173450

**Published:** 2017-03-09

**Authors:** Anna-Maija Tolppanen, Ari Voutilainen, Heidi Taipale, Antti Tanskanen, Piia Lavikainen, Marjaana Koponen, Jari Tiihonen, Sirpa Hartikainen

**Affiliations:** 1 School of Pharmacy, University of Eastern Finland, Kuopio, Finland; 2 Research Centre for Comparative Effectiveness and Patient Safety (RECEPS), University of Eastern Finland, Kuopio, Finland; 3 Department of Nursing Science, University of Eastern Finland, Kuopio, Finland; 4 Kuopio Research Centre of Geriatric Care, University of Eastern Finland, Kuopio, Finland; 5 Department of Forensic Psychiatry, Niuvanniemi Hospital, Kuopio, Finland; 6 Department of Clinical Neuroscience, Karolinska Institutet, Stockholm, Sweden; 7 National Institute for Health and Welfare, Helsinki, Finland; 8 Department of Psychiatry, Kuopio University Hospital, Kuopio, Finland; Istituto Di Ricerche Farmacologiche Mario Negri, ITALY

## Abstract

**Objectives:**

To describe and compare temporal changes in prevalence and incidence of psychotropic use (antipsychotics, antidepressants and benzodiazepines and related drugs; BZDRs) in persons with newly diagnosed Alzheimer’s disease (AD) between university hospital districts of Finland during 2005–2011.

**Methods:**

The MEDALZ study includes all community-dwellers of Finland who received a clinically verified AD diagnosis in 2005–2011 (N = 70,718). Prevalent and incident use of psychotropics among those who had received AD diagnosis less than one year ago were compared in 2005–2011.

**Results:**

Regional differences in psychotropic use between university hospital districts were more evident in 2005 than 2011 for prevalent use of any psychotropic, antipsychotic and BZDRs and incident use of any psychotropic and antipsychotics. Regional differences in prevalent antidepressant use and incident BZDR use remained similar during the follow-up, while differences in incident antidepressant use increased during the follow-up. The prevalence of any psychotropic use in 2005 varied between 44.7–50.7% and between 45.0–47.9% in 2011. Incidence of any psychotropic use in 2005 was between 8.6–12.1% and 6.2–8.2% in 2011. In 2005, the distribution of incident psychotropic use followed a large scale spatial variation that, however, did not correspond to university hospital districts. During the study period from 2005 to 2011 the cyclic spatial variation disappeared. No sign of adjacent hospital districts being more or less closely related to each other compared to hospital districts in general was detected.

**Conclusions:**

Except for antidepressants, regional differences in psychotropic use have mainly diminished between 2005 and 2011. Our findings highlight the importance of acknowledging regional differences in a country with relatively homogeneous healthcare system and conducting future studies assessing the reasons behind these differences.

## Introduction

Although antidementia medications (acetylcholinesterase inhibitors and memantine) are recommended as the first-line treatments for behavioural and psychological symptoms of dementia, [1] psychotropic drugs (antidepressants, antipsychotics and benzodiazepines and related drugs; BZDRs) are frequently used among persons with dementia or Alzheimer’s disease (AD; the most common form of dementia). Studies on psychotropic use among persons with incident dementia/AD have reported an annual prevalence of 13–31% for antipsychotic use, 22–54% for antidepressants and 5–29% for BZDRs (depending on the definition of BZDRs).[2–7] These numbers are considerably higher than in age- and sex-matched population without AD/dementia [3,4,6,7] and have remained high despite the safety warnings.[2–5,7]

Results of longitudinal studies assessing the temporal change have been different depending on the study period, setting and participants. A German study did not detect any change in overall prevalence of antipsychotic use among persons with dementia during 2004–2009,[5] while in Italy a decline in overall antipsychotic use among acetylcholinesterase inhibitor users was reported during 2002–2008, despite concomitant rise in atypical antipsychotic use.[2] In an UK study a decrease in antipsychotic and hypnotics use during 1995–2005 occurred among dementia patients, but this was offset by an increase in antidepressant and anxiolytic use.[3] In Denmark antidepressant use increased while the use of antipsychotics, anxiolytics and hypnotics decreased in 2000–2012 among persons with dementia.[7] In our previous study including all community-dwellers who received a clinically verified AD diagnosis in Finland during 2005–2011, we detected an increasing trend of antipsychotic use, no changes in antidepressant use and decline in BZDR use.[4] Our study on the regional differences of the same cohort showed regional variation in antidementia medication use across university hospital districts,[8] but it is not known if similar regional variation exists in psychotropic use.

### Aims of the study

Aims of the study were to describe and compare regional differences in the temporal changes of incidence and prevalence of psychotropic use in persons with newly diagnosed AD in Finland during the years 2005–2011.

## Material and methods

### Study cohort

The Medication and Alzheimer’s disease (MEDALZ) cohort includes all community-dwelling persons who received a clinically verified diagnosis of AD in 2005–2011 (N = 70,718). The age range of the cohort was 34–105 years (mean 80.1 (SD 7.1) years) and 65.2% of the study population were women. The cohort has been described previously in more detail.[9] Persons with AD were identified from the Finnish Special Reimbursement Register maintained by the Social Insurance Institution of Finland (SII). The Special Reimbursement Register contains records of all persons who are eligible for higher reimbursement due to certain chronic diseases, including AD. To be eligible for reimbursement, the disease must be diagnosed according to specific criterion and diagnosis statement must be submitted to the SII by a physician. The AD diagnosis was mainly based on the NINCDS-ADRDA and DSM-IV criteria for Alzheimer’s disease.[10,11] Briefly, the criterion for AD includes 1) symptoms consistent with mild or moderate AD, 2) decrease in social capacity over a period of at least 3 months, 3) computed tomography/magnetic resonance imaging scan, 4) exclusion of possible alternative diagnoses, and 5) confirmation of the diagnosis by a registered geriatrician or neurologist. The main difference to the 1984 version of NINCDS-ADRDA is that the special reimbursement criterion requires nontransient decline in social capacity and does not set a specific age limit for diagnosis. In addition, confirmation of diagnosis by a geriatrician/ neurologist and a CT scan or MRI were not required in either NINCDS-ADRDA or DSM-IV criteria. A summary of anamnestic information from the patients and family, as well as findings e.g. MRI/CT, laboratory tests, CERAD, are submitted to the SII, where a geriatrician/neurologist systematically evaluates the diagnostic evidence for each AD case and confirms whether the pre-specified criteria are met.

Each resident of Finland is assigned a unique personal identity code which was used to compile the research database from various national registers as described previously.[12] All data were de-identified by the register maintainers and ethics committee approval or informed consent were not required as only de-identified data were used and the study participants were not contacted.

In Finland, primary care is organised by municipalities. Each municipality belongs to one of the 21 hospital districts that are responsible for organising specialised healthcare and ensuring that it complements the primary care. (http://stm.fi/en/social-and-health-services/responsible-agencies) The most advanced specialised medical care is organised by five university hospital districts (Helsinki, Turku, Tampere, Oulu and Kuopio), that are formed by the hospital districts. The health care services are implemented with government support according to the legislation and monitored by the Ministry of Social Affairs and Health.

### Medication use

Data on all prescribed medications dispensed between 1995 and 2011 were extracted from the Finnish National Prescription Register which covers reimbursed prescription purchases, with the exception of those provided during stays at hospitals and public nursing homes. All medications in the Prescription Register are categorised according to the World Health Organization (WHO) Anatomical Therapeutic Chemical (ATC) Classification system and purchased amounts are recorded in Defined Daily Doses (DDDs); the assumed average maintenance dose per day for a drug used for its main indication in adults.[13]http://bjp.rcpsych.org/content/207/5/444.long—ref-26 For each person and ATC code, use periods were modelled with a validated PRE2DUP method.[14] PRE2DUP is based on sliding averages of DDD and it accounts for hospitalisations, stockpiling and dose changes.

Antipsychotics were identified by ATC code N05A (excluding lithium and prochlorperazine), antidepressants by ATC code N06A and benzodiazepines and benzodiazepine-related drugs by ATC codes N05BA, N05CD and N05CF. Use of antidementia medication (ATC code N06D) was modelled similarly and only prevalent use was considered. None of the cohort members had antidementia medication purchases before AD diagnosis so the incidence corresponds to prevalence (except for those persons who were diagnosed on the previous calendar year and had purchased these medications already on the diagnosis year).

### Statistical analyses

Statistical analyses were performed with Stata/MP 14.1, R and IBM SPSS Statistics 21. In each calendar year, we investigated the prevalent and incident use of psychotropics among persons with newly diagnosed AD, i.e., those who had at maximum one year since their AD diagnosis. Eligibility for prevalence analyses was defined as 1) AD diagnosis not more than one year ago 2) not hospitalised/institutionalised for the entire assessment period, as medication use during hospitalisation or institutionalisation cannot be ascertained from the registers. For incidence analyses, one-year washout period was used, i.e., persons who had used the drug of interest one year before AD diagnosis were not eligible. The prevalence and incidence data are presented as percentage of users of eligible population, as well as age- and sex-standardised rates (per 100 person-years). The follow-up calculation for rates was terminated on the date of death or admission date of over 90-day hospitalisation or institutionalisation if this occurred earlier than the end of one-year follow-up period. Differences in changes in proportions were compared with χ^2^-test and differences in age with t-test.

Multilevel mixed effects linear regression models were fit to 1) investigate temporal changes in the age- and sex adjusted rates 2) assess whether the slope was different between hospital or university hospital districts 3) quantify whether the variation was larger between university hospital districts or hospital districts. Time was included as a fixed effect and hospital and university hospital districts as nested random effects. Models were fitted with unstructured covariance matrix (i.e., all variances and covariances were estimated separately). In addition, we investigated whether antidementia medication use was associated with psychotropic use by including antidementia medication use as a fixed effect in these models.

In addition, principal coordinates of neighbour matrices (PCNM) were created to detect the spatial scale which had the strongest effect on the incidence of psychotropic use. PCNM is based on geographical distances between different sites.[15] It produces variables, spatial vectors, which correspond to autocorrelation and model spatial relationships between the sites; the larger the vector score, the larger the scale of the cyclic variation. In PCNM, cyclic variation represents regular geographic patterns with various spatial scales. Small-scale variation means that the study area is composed of numerous similar, regularly repeated, patterns and, correspondingly, large-scale variation means that the study area is composed of only a few patterns. A pattern corresponds to a wave with one crest (high values) and one trough (low values). The method and its epidemiological applications have been described in detail by Voutilainen et al.[16,17] In the present study, latitudes and longitudes of the largest city in each hospital district were used as initial values for PCNM. Another option would have been to use geographic centres of hospital districts as initial values but largest cities were though to better represent people living in the areas. In general, this kind of analysis procedure is typical and suitable for finding answers for epidemiological research questions.[16,17] Aland (an archipelago) was excluded from the analyses due to its restricted connections with other 20 hospital districts. Then, the spatial vectors created by PCNM from these geographical locations were used as independent variables in a linear regression model with psychotropic incidence rate as a dependent variable. PCNM vectors were created using functions of the ‘vegan’ package for R.[18]

## Results

### Participant characteristics in different university hospital districts

Table 1 summarises the demographic characteristics and prevalence of psychotropic use in different university hospital districts in 2005 and 2011. More detailed description including data from years 2006–2010 and from different hospital districts is available in S1 Table and S2 Table. The proportion of women was similar across university hospital districts, varying between 62.5–66.8% in 2005 and 63.0–65.4 in 2011 and changes in the sex distribution in different districts were not observed during the study period (Table 1). In 2005, the annual cohort of Oulu was on average one year younger than the Turku cohort, which had the highest average age, 79.9 years in 2005. The average age increased by approximately one year in all districts between 2005 and 2011 and the differences between university hospital districts remained. Regional differences in the use of antidementia medication were evident in both 2005 and 2011. Altogether 63.8–64.9% of persons who were diagnosed with AD in 2005 and resided in Kuopio, Oulu or Tampere districts purchased antidementia medication in that year, while the proportions in Turku and Helsinki were somewhat smaller, 55.8% and 57.3, respectively. By 2011, the differences had narrowed as the proportion of those with antidementia medication purchases was 73.4–78% for all university hospital districts, although the lowest prevalences were still observed in Helsinki and Turku.

**Table 1:** Demographic characteristics and prevalence of psychotropic use in different university hospital districts in 2005 and 2011. Data are given as n (%) unless otherwise indicated.

	2005		2011		P for temporal change
	N (n eligible*)		N (n eligible*)		
Age, mean (SD)					
Helsinki	2,126 (2,104)	79.5 (6.9)	6,868 (6,614)	80.4 (7.6)	<0.001
Kuopio	1,673 (1,654)	79.3 (6.8)	4,790 (4,612)	80.3 (6.9)	<0.001
Oulu	1,350 (1,342)	78.9 (6.5)	3,117 (3,054)	80.3 (7.1)	<0.001
Tampere	1,961 (1,942)	79.4 (6.7)	5,013 (4,820)	80.6 (7.1)	<0.001
Turku	1,410 (1,397)	79.9 (6.5)	3,249 (3,159)	81.1 (7.1)	<0.001
P for regional difference		0.002		<0.001	
Sex, women					
Helsinki	2,126 (2,104)	1,398 (66.4)	6,868 (6,614)	4,314 (65.2)	0.31
Kuopio	1,673 (1,654)	1,055 (63.8)	4,790 (4,612)	2,947 (63.9)	0.93
Oulu	1,350 (1,342)	839 (62.5)	3,117 (3,054)	1,924 (63.0)	0.76
Tampere	1,961 (1,942)	1,269 (65.4)	5,013 (4,820)	3,065 (63.6)	0.17
Turku	1,410 (1,397)	933 (66.8)	3,249 (3,159)	2,066 (65.4)	0.36
P for regional difference		0.07		0.10	
Antidementia medication use					
Helsinki	2,126 (2,104)	1,205 (57.3)	6,868 (6,614)	4,867 (74.6)	<0.001
Kuopio	1,673 (1,654)	1, 055 (63.8)	4,790 (4,612)	3,596 (78.0)	<0.001
Oulu	1,350 (1,342)	853 (63.6)	3,117 (3,054)	2,348 (76.9)	<0.001
Tampere	1,961 (1,942)	1,260 (64.9)	5,013 (4,820)	3,690 (76.6)	<0.001
Turku	1,410 (1,397)	780 (55.8)	3,249 (3,159)	2,317 (73.4)	<0.001
P for regional difference		<0.001		<0.001	
Any psychotropic, prevalent use					
Helsinki	2,126 (2,104)	1,067 (50.7)	6,868 (6,614)	3,064 (46.3)	<0.001
Kuopio	1,673 (1,654)	767 (46.4)	4,790 (4,612)	2,076 (45.0)	0.34
Oulu	1,350 (1,342)	600 (44.7)	3,117 (3,054)	1,377 (45.1)	0.82
Tampere	1,961 (1,942)	952 (49.0)	5,013 (4,820)	2,292 (47.6)	0.27
Turku	1,410 (1,397)	690 (49.4)	3,249 (3,159)	1,512 (47.9)	0.34
P for regional difference		0.004		0.027	
Antipsychotics, prevalent use					
Helsinki	2,126 (2,104)	341 (16.2)	6,868 (6,614)	1,043 (15.8)	0.63
Kuopio	1,673 (1,654)	303 (18.3)	4,790 (4,612)	789 (17.1)	0.27
Oulu	1,350 (1,342)	173 (12.9)	3,117 (3,054)	490 (16.0)	0.007
Tampere	1,961 (1,942)	301 (15.5)	5,013 (4,820)	820 (17.0)	0.13
Turku	1,410 (1,397)	224 (16.0)	3,249 (3,159)	554 (17.5)	0.21
P for regional difference		0.002		0.12	
Antidepressants, prevalent use					
Helsinki	2,126 (2,104)	595 (28.3)	6,868 (6,614)	1,775 (26.8)	0.20
Kuopio	1,673 (1,654)	368 (22.3)	4,790 (4,612)	1,031 (22.4)	0.93
Oulu	1,350 (1,342)	316 (23.6)	3,117 (3,054)	693 (22.7)	0.54
Tampere	1,961 (1,942)	491 (25.3)	5,013 (4,820)	1,192 (24.7)	0.63
Turku	1,410 (1,397)	368 (26.3)	3,249 (3,159)	768 (24.3)	0.14
P for regional difference		<0.001		<0.001	
BZDR, prevalent use					
Helsinki	2,126 (2,104)	596 (28.3)	6,868 (6,614)	1,535 (23.2)	<0.001
Kuopio	1,673 (1,654)	433 (26.2)	4,790 (4,612)	1,038 (22.5)	0.003
Oulu	1,350 (1,342)	376 (28.0)	3,117 (3,054)	731 (23.9)	0.004
Tampere	1,961 (1,942)	604 (31.1)	5,013 (4,820)	1,202 (24.9)	<0.001
Turku	1,410 (1,397)	399 (28.6)	3,249 (3,159)	770 (24.4)	0.003
P for regional difference		0.027		0.050	

*AD diagnosis not more than one year ago and not hospitalised/institutionalised for the entire assessment period

### Prevalent psychotropic use

**Fig 1:**
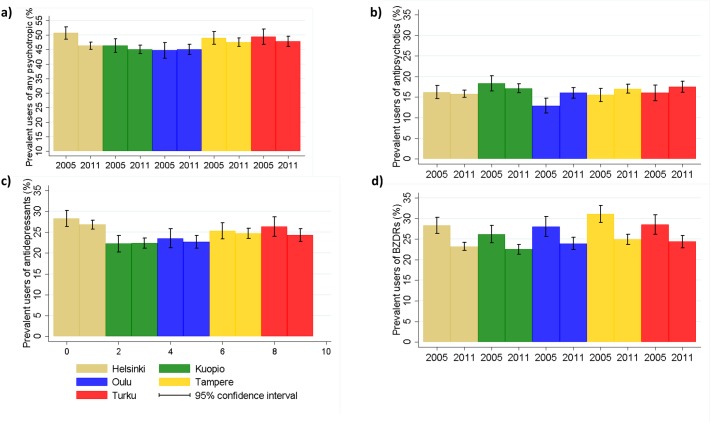
The prevalence of a) any psychotropic b) antipsychtotic c) antidepressant and d) benzodiazepines and related drug (BZDR) use in different university hospital regions in 2005 and 2011.

The prevalence of any psychotropic use in 2005 varied from 44.7% (Oulu) to 50.7% (Helsinki; Table 1, Fig 1). Prevalent use of antipsychotics in 2005 was most common in Kuopio (18.3%) and least common in Oulu (12.9%), while antidepressant and BZDR use was least prevalent in Kuopio (22.3% and 26.2%, respectively). Highest prevalence of antidepressant use in 2005 was observed in Helsinki (28.3%) and prevalent BZDR use was most common in Tampere (31.1%). Thus, the prevalence of any psychotropic use, as well as the use of individual psychotropics varied between university hospital districts in 2005. The differences were less evident in 2011 for BZDR use, which had declined similarly in all university hospital districts. Regional differences in antipsychotic use disappeared by 2011, mainly because the prevalence increased in the Oulu district but remained similar in other regions. The prevalence of antidepressant use remained similar in all districts and thus, the regional differences observed in 2005 were evident also in 2011. The changes in individual categories largely complemented each other in Oulu, Kuopio, Tampere and Turku university hospital districts, where no overall change in the prevalence of any antipsychotic use was observed between 2005–2011 while in Helsinki the prevalence decreased by 4.4%. The change in prevalence rates is illustrated in more detail in S1 Movie and S2 Movie which display the annual prevalence of any psychotropic use in different university hospital districts and hospital districts.

### Incident psychotropic use

**Fig 2:**
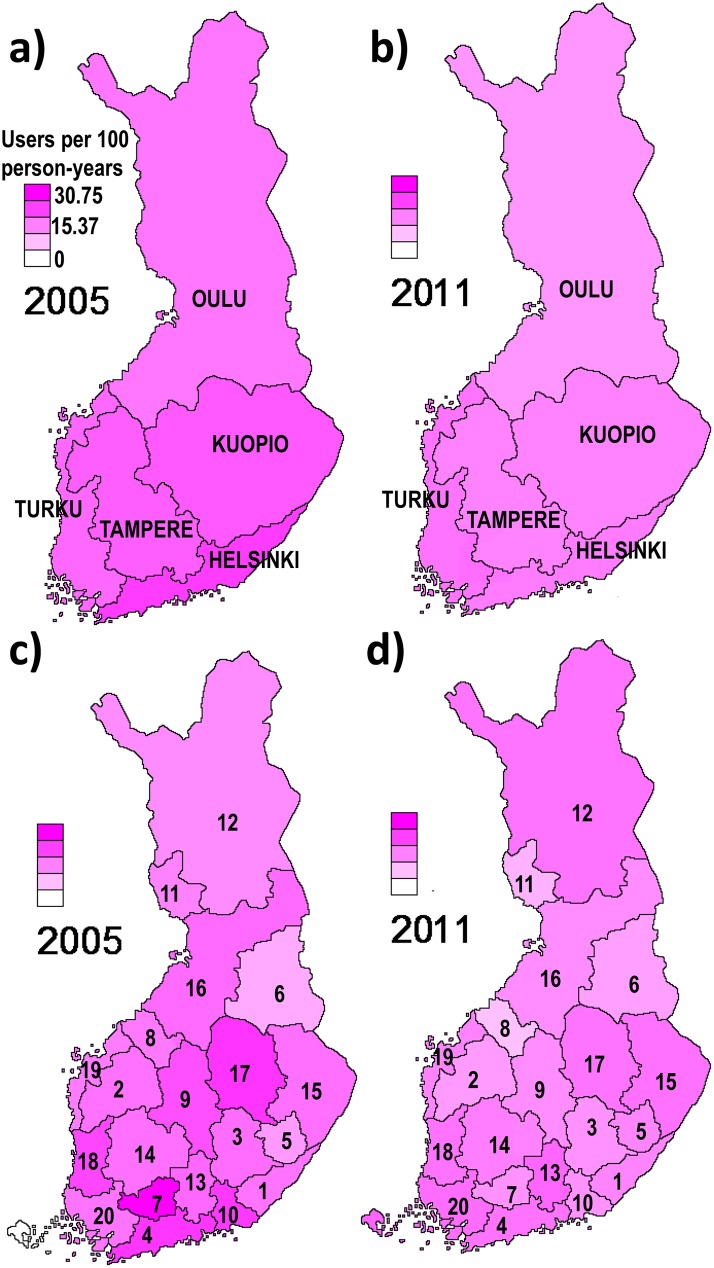
Incidence of any psychotropic use in different university hospital districts (a-b) and hospital districts (c-d; numbers correspond to those in S2 Table) in 2005 and 2011.

**Fig 3:**
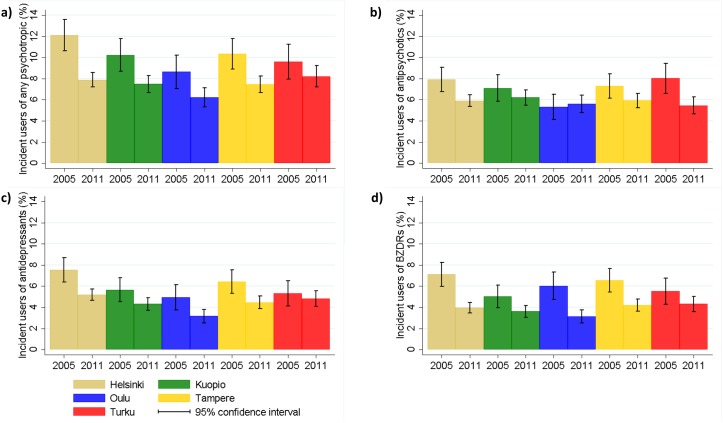
The incidence of a) any psychotropic b) antipsychtotic c)antidepressant and d) benzodiazepines and related drug (BZDR) use in different university hospital regions in 2005 and 2011.

Regional differences between university hospital districts were observed also for incident psychotropic use in 2005, but these differences were less evident in 2011 for other drug groups except for antidepressants (Table 2). In 2005, the incidence rates in the hospital districts within each university hospital district differed more from each other than in 2011 (Figs 2 and 3). In 2005 the lowest incidence was observed in Oulu for any psychotropic, antipsychotic and antidepressant use (8.6%, 5.3% and 4.9%, respectively) and the highest in Helsinki for any psychotropic (12.1%) and antidepressants (7.5%) and in Turku for antipsychotics (8.0%). However, the differences between districts were smaller than for prevalence. In 2011, there was less variation between university hospital districts: the incidence of any psychotropic use was 6.2–8.2%, and the incidence of antipsychotics, antidepressants and BZDRs were 5.5–6.2%, 3.2–5.2% and 3.1–4.3%, respectively. As with prevalence, the largest decrease in overall psychotropic use occurred in Helsinki where the incidence in 2005 was the highest. Similar results were observed with age and sex-adjusted incidence rates which account for demographic differences between AD cases in different districts. The annual changes in incidence rates in different university hospital districts are displayed in supplementary material for any psychotropics (S3 Movie), antipsychotics (S4 Movie), antidepressants (S5 Movie) and BZDRs (S6 Movie).

**Table 2:** Incidence of psychotropic use in different university hospital districts in 2005 and 2011.

	2005 n (%)	2011 n (%)	P for temporal change	2005 incidence rate/100PY	2011 incidence rate/100PY
Any psychotropic, incident use					
Helsinki	223 (12.1)	463 (7.9)	<0.001	23.5	15.9
Kuopio	151 (10.2)	314 (7.5)	0.001	20.1	15.1
Oulu	104 (8.6)	171 (6.2)	0.006	16.5	12.7
Tampere	177 (10.4)	324 (7.5)	<0.001	19.5	15.1
Turku	119 (9.6)	236 (8.2)	0.15	18.1	16.8
P for regional difference	0.029	0.045			
Antipsychotics, incident use					
Helsinki	164 (7.9)	384 (5.9)	0.001	14.7	11.7
Kuopio	115 (7.1)	282 (6.2)	0.21	13.2	12.2
Oulu	70 (5.3)	168 (5.6)	0.69	10.0	11.2
Tampere	139 (7.3)	281 (5.9)	0.038	15.0	11.7
Turku	110 (8.0)	169 (5.5)	0.001	15.1	10.9
P for regional difference	0.040	0.69			
Antidepressants, incident use					
Helsinki	153 (7.5)	331 (5.2)	<0.001	14.0	10.2
Kuopio	91 (5.7)	194 (4.3)	0.028	10.9	8.5
Oulu	64 (4.9)	84 (3.2)	0.005	8.7	6.4
Tampere	121 (6.4)	209 (4.5)	0.001	11.7	9.0
Turku	72 (5.3)	149 (4.8)	0.49	9.5	9.7
P for regional difference	0.013	<0.001			
BZDR, incident use					
Helsinki	142 (7.1)	252 (4.0)	<0.001	13.7	7.8
Kuopio	80 (5.0)	161 (3.6)	0.014	9.4	7.2
Oulu	78 (6.0)	92 (3.1)	<0.001	11.0	6.3
Tampere	122 (6.5)	196 (4.2)	<0.001	13.8	8.4
Turku	74 (5.5)	132 (4.3)	0.08	10.1	8.7
P for regional difference	0.08	0.08			

**Fig 4:**
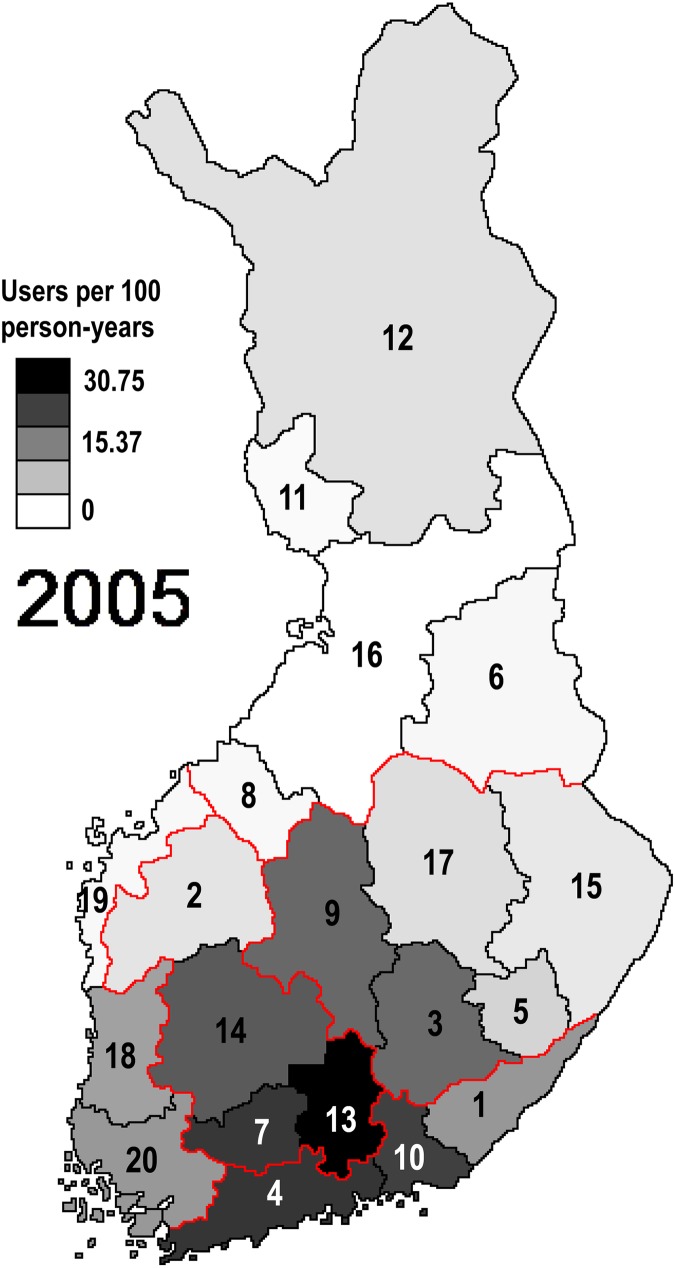
Modelled spatial distribution of incident psychotropic use in 2005. The model explained circa 30% of the actual distribution (see Fig 2). Red lines indicate borders of university hospital districts and numbering of hospital districts corresponds to S2 Table.

In 2005, the incidence of any psychotropic use followed a large scale spatial distribution (Fig 4). A single spatial vector distributing Finland into approximately five areas explained 33.5% of the observed variation in psychotropic use. The spatial vector, however, did not correspond to five university hospital districts. During the study period, from 2005 to 2011, the cyclic spatial distribution of psychotropic use disappeared so that, in 2011, spatial vectors were unable to explain any of the variation in psychotropic use. According to PCNM, there was no evidence for adjacent hospital districts being more or less related to each other compared to hospital districts in general.

Multilevel mixed effects linear regression models provided similar results (Table 3). Variation in the temporal change in prevalence or incidence of psychotropic use was explained more by hospital districts than university hospital districts. However, it should be noted that in most cases the residual variance was larger than explained variance. There was no evidence for different slopes for university hospital or hospital districts, i.e., similar changes occurred in all districts.

**Table 3:** Coefficients and 95% confidence intervals for random effect parameters university hospital and hospital district and antidementia medication use (fixed effect).

	University hospital district	Hospital district	Residual variance	Antidementia medication
Prevalence				
Any psychotropic	0	30.26 (14.75–62.09)	33.45 (25.98–43.09)	-0.83 (-1.42, -0.24)
Antipsychotics	1.42 (0.07–29.51)	6.44 (2.4–16.85)	16.82 (13.06–21.66)	0.17 (-0.40, 0.74)
Antidepressants	0	45.67 (23.63–88.26)	20.08 (15.60–25.87)	-0.58 (-1.11, -0.06)
BZDRs	0	28.71 (14.58–56.51)	18.52 (14.38–23.85)	0.25 (-0.40, 0.90)
Incidence				
Any psychotropic	0.62 (0.07–5.55)	1.13 (0.30–4.26)	6.89 (5.34–8.87)	-0.36 (-0.70, -0.02)
Antipsychotics	0.22 (0.01–7.85)	1.09 (0.34–4.53)	5.18 (4.01–6.66)	0.13 (-0.17, 0.44)
Antidepressants	0.39 (0.02–6.31)	1.46 (0.55–3.90)	4.02 (3.12–5.18)	-0.33 (-0.58, -0.08)
BZDRs	0	0.48 (0.10–2.32)	4.95 (3.84–6.37)	-0.04 (-0.28, 0.20)

Use of antidementia medication was negatively associated with antidepressant use, as consequently with any psychotropic use (Table 3). No associations with antipsychotic or BZDR use were observed.

## Discussion

Our nationwide study among recently diagnosed AD cases showed that the differences in incident psychotropic use between university hospital districts mainly disappeared or diminished between 2005 and 2011, except for antidepressants. However, also for antidepressants the absolute difference between the highest and the lowest incidence was only 3.8 initiations/100 person-years. These incidence changes were also reflected in regional prevalence differences, which disappeared altogether for antipsychotics and were decreased for BZDRs and any psychotropic drug but remained on the same level for antidepressants.

The lowest prevalence and incidence rates for almost all psychotropic groups in all study years were observed in the Oulu district, which together with the Kuopio district had the highest rate of antidementia medication purchases within one year of AD diagnosis.[8] The prevalence rates of any psychotropic and antidepressants, and prevalence and incidence of BZDR use were also lower in Kuopio in comparison to Turku, Tampere and Helsinki districts. The largest decreases were often observed in those university hospital districts with the highest incidence or prevalence in 2005.

Interestingly, the Helsinki district where the incidence and prevalence rates of many psychotropic classes were the highest or among the highest, had the lowest incidence of antidementia medication purchases in our previous study.[8] Similarly, we observed a negative association between antidementia medication and psychotropic use in the present study, i.e. the use of psychotropics, especially antidepressants was less frequent in those districts where the use of antidementia medications was most common. These findings may reflect differences in how the healthcare systems of different districts handle persons with AD. However, it should be acknowledged that our data cannot be used for assessing the reasons behind these differences, but we hope to see future studies in this topic. Differences in the used antidementia medications (acetylcholinesterase inhibitors vs memantine) were not reflected in our study, as the district with highest proportion of memantine users (Tampere) [8] did not display large differences in psychotropic use in comparison to other university hospital districts.

The disappearance of incidence differences during 2005–2011 may reflect changes in treatment practices. It is possible that some university hospital districts had lower threshold for prescribing psychotropics for behavioural and psychological symptoms of dementia, or that persons received the AD diagnosis at later stage of disease in those university hospital districts with the highest prevalence/incidence of psychotropic use. It is difficult to assess the threshold for prescribing psychotropics, but the number of persons who are eligible for special reimbursement to AD has increased by 43% between 2005–2011,[9] and the numbers from 2011 are relatively close to the estimated number of incident AD cases in Finland (13,000).[19]

The overall incidence of psychotropic use decreased during the follow-up in all university hospital districts. This may be perceived as a positive sign from the safety perspective. However, our previous studies indicate that psychotropics are frequently initiated already before the AD diagnosis.[20–22] According to our incidence definition, person who had been using psychotropics one year before AD diagnosis were excluded as the focus is on new initiators. Thus, another possible explanation for the observed decreasing incidence in this study is that psychotropic medications are initiated for prodromal neuropsychiatric symptoms already before the AD diagnosis. This explanation is partly supported by our finding that decreases in prevalence of psychotropic use occurred in a smaller scale.

Strengths of our study include the nationwide cohort of persons with clinically verified AD diagnosis. However, as the sample was restricted to those who were community-dwelling at the beginning of follow-up, the results are not generalisable to institutionalised persons. This restriction was applied because medications provided at certain institutions and in all hospitals are not recorded in the prescription register and thus inclusion of institutionalised persons would have increased the possibility of misclassification bias. For the same reason, the follow-up was discontinued at long-term hospitalisation or institutionalisation. The psychotropic purchase data were obtained from national registers, which include all purchased reimbursable medications. As small packages of BZDRs are not reimbursed we cannot exclude the possibility that decreasing trend in BZDR use may reflect a shift towards smaller packages instead of actual decrease in use. Although purchased medications may not always reflect consumed medications, our results are not prone to recall bias, and the dispensing data approximates the medication use better than prescription data.[23]

In summary, a similar decrease occurred in the incidence and prevalence of psychotropic use in all university hospital and hospital districts of Finland in 2005–2011, but regional difference in antidepressant incidence and prevalence was still evident in 2011. In addition, the overall proportion of prevalent users was still high in 2011. It would be important to assess the reasons behind regional differences.

## Supporting information

S1 Table Incidence and prevalence of psychotropic use in university hospital districts in 2005-2011. Rates are given as users/100 person-years. BZDRs Benzodiazepines and related drugs.
(DOCX)

S2 Table Incidence and prevalence of psychotropic use in hospital districts in 2005-2011. Rates are given as users/100 person-years. BZDRs Benzodiazepines and related drugs. Numbers in the hospital district correspond to those in Fig 2
(DOCX)

S1 Movie The annual prevalence of any psychotropic use in different university hospital districts. (MP4)

S2 Movie The annual prevalence of any psychotropic use in different hospital districts. (MP4)

S3 Movie The annual changes in incidence of any psychotropic use in different university hospital districts. (MP4)

S4 Movie The annual changes in incidence of antipsychotic use in different university hospital districts. (MP4)

S5 Movie The annual changes in incidence of any antidepressant use in different university hospital districts. (MP4)

S6 Movie The annual changes in incidence of benzodiazepine and related drug (BZDR) use in different university hospital districts. (MP4)
