# Supplementary material for: Regional changes in psychotropic use among Finnish persons with newly diagnosed Alzheimer’s disease in 2005-2011
Source: PLoS One. 2017 Mar 9;12(3):e0173450. doi: 10.1371/journal.pone.0173450 (PMC5344395; doi:10.1371/journal.pone.0173450)
Supplement: S1 Table — Rates are given as users/100 person-years. BZDRs Benzodiazepines and related drugs. (DOCX) [file pone.0173450.s001.docx]

S1 Table Incidence and prevalence of psychotropic use in university hospital districts in 2005-2011.Rates are given as users/100 person-years. BZDRs Benzodiazepines and related drugs.

| District | Year | Drug | Incidence | | | | Prevalence | | | |
| --- | --- | --- | --- | --- | --- | --- | --- | --- | --- | --- |
|  |  |  | n of users | n of eligible persons | % of users | Age- and sex-adjusted rate | n of users | n of eligible persons | % of users | Age- and sex-adjusted rate |
| Helsinki | 2005 | Any | 223 | 1,841 | 12.1 | 23.5 | 1,067 | 2,104 | 50.7 | 93.8 |
|  | 2006 |  | 330 | 3,762 | 8.8 | 18 | 2,179 | 4,272 | 51 | 101.3 |
|  | 2007 |  | 316 | 4,167 | 7.6 | 15.8 | 2,334 | 4,693 | 49.7 | 99.3 |
|  | 2008 |  | 450 | 4,578 | 9.8 | 19.5 | 2,622 | 5,173 | 50.7 | 97.7 |
|  | 2009 |  | 380 | 4,815 | 7.9 | 16.6 | 2,764 | 5,485 | 50.4 | 102.8 |
|  | 2010 |  | 422 | 5,154 | 8.2 | 16.8 | 2,868 | 5,847 | 49.1 | 97.1 |
|  | 2011 |  | 463 | 5,876 | 7.9 | 15.9 | 3,064 | 6,614 | 46.3 | 90.4 |
|  | 2005 | Antipsychotics | 164 | 2,072 | 7.9 | 14.7 | 341 | 2,104 | 16.2 | 29.8 |
|  | 2006 |  | 255 | 4,206 | 6.1 | 12 | 700 | 4,272 | 16.4 | 32.4 |
|  | 2007 |  | 222 | 4,628 | 4.8 | 9.7 | 684 | 4,693 | 14.6 | 29.1 |
|  | 2008 |  | 362 | 5,082 | 7.1 | 13.9 | 880 | 5,173 | 17 | 32.7 |
|  | 2009 |  | 325 | 5,385 | 6 | 12.3 | 950 | 5,485 | 17.3 | 35.1 |
|  | 2010 |  | 343 | 5,749 | 6 | 11.9 | 978 | 5,847 | 16.7 | 33 |
|  | 2011 |  | 384 | 6,496 | 5.9 | 11.7 | 1,043 | 6,614 | 15.8 | 30.7 |
|  | 2005 | Antidepressants | 153 | 2,031 | 7.5 | 14 | 595 | 2,104 | 28.3 | 52.1 |
|  | 2006 |  | 229 | 4,127 | 5.5 | 11.1 | 1,248 | 4,272 | 29.2 | 57.3 |
|  | 2007 |  | 236 | 4,539 | 5.2 | 10.5 | 1,372 | 4,693 | 29.2 | 57.8 |
|  | 2008 |  | 279 | 5,010 | 5.6 | 10.8 | 1,483 | 5,173 | 28.7 | 54.9 |
|  | 2009 |  | 291 | 5,298 | 5.5 | 11.3 | 1,583 | 5,485 | 28.9 | 58.7 |
|  | 2010 |  | 284 | 5,610 | 5.1 | 10.1 | 1,649 | 5,847 | 28.2 | 55.7 |
|  | 2011 |  | 331 | 6,378 | 5.2 | 10.2 | 1,775 | 6,614 | 26.8 | 52.3 |
|  | 2005 | BZDRs | 142 | 1,996 | 7.1 | 13.7 | 596 | 2,104 | 28.3 | 52.6 |
|  | 2006 |  | 211 | 4,092 | 5.2 | 10.4 | 1,209 | 4,272 | 28.3 | 56.6 |
|  | 2007 |  | 226 | 4,490 | 5 | 10.2 | 1,303 | 4,693 | 27.8 | 55.7 |
|  | 2008 |  | 271 | 4,982 | 5.4 | 10.6 | 1,392 | 5,173 | 26.9 | 52.2 |
|  | 2009 |  | 224 | 5,246 | 4.3 | 8.8 | 1,410 | 5,485 | 25.7 | 52.7 |
|  | 2010 |  | 256 | 5,603 | 4.6 | 9.1 | 1,488 | 5,847 | 25.4 | 50.7 |
|  | 2011 |  | 252 | 6,370 | 4 | 7.8 | 1,535 | 6,614 | 23.2 | 45.4 |
| Kuopio | 2005 | Any | 151 | 1,478 | 10.2 | 20.1 | 767 | 1,654 | 46.4 | 86.4 |
|  | 2006 |  | 273 | 2,988 | 9.1 | 19.6 | 1,567 | 3,308 | 47.4 | 98.3 |
|  | 2007 |  | 265 | 3,217 | 8.2 | 16.4 | 1,742 | 3,620 | 48.1 | 93.6 |
|  | 2008 |  | 299 | 3,609 | 8.3 | 16.9 | 1,947 | 4,038 | 48.2 | 94.5 |
|  | 2009 |  | 296 | 3,835 | 7.7 | 16.2 | 2,034 | 4,247 | 47.9 | 97.9 |
|  | 2010 |  | 288 | 3,836 | 7.5 | 15.1 | 1,998 | 4,271 | 46.8 | 92.1 |
|  | 2011 |  | 314 | 4,193 | 7.5 | 15.1 | 2,076 | 4,612 | 45 | 88.7 |
|  | 2005 | Antipsychotics | 115 | 1,620 | 7.1 | 13.2 | 303 | 1,654 | 18.3 | 34.2 |
|  | 2006 |  | 192 | 3,250 | 5.9 | 12.7 | 593 | 3,308 | 17.9 | 37.7 |
|  | 2007 |  | 219 | 3,556 | 6.2 | 12.2 | 633 | 3,620 | 17.5 | 34 |
|  | 2008 |  | 272 | 3,966 | 6.9 | 13.6 | 746 | 4,038 | 18.5 | 36.2 |
|  | 2009 |  | 252 | 4,180 | 6 | 12.3 | 788 | 4,247 | 18.6 | 38 |
|  | 2010 |  | 272 | 4,196 | 6.5 | 12.8 | 784 | 4,271 | 18.4 | 36.2 |
|  | 2011 |  | 282 | 4,548 | 6.2 | 12.2 | 789 | 4,612 | 17.1 | 33.6 |
|  | 2005 | Antidepressants | 91 | 1,609 | 5.7 | 10.9 | 368 | 1,654 | 22.2 | 41.6 |
|  | 2006 |  | 162 | 3,226 | 5 | 10.2 | 744 | 3,308 | 22.5 | 46 |
|  | 2007 |  | 173 | 3,519 | 4.9 | 9.5 | 870 | 3,620 | 24 | 46.6 |
|  | 2008 |  | 182 | 3,935 | 4.6 | 9.3 | 967 | 4,038 | 23.9 | 46.9 |
|  | 2009 |  | 178 | 4,160 | 4.3 | 8.8 | 1,010 | 4,247 | 23.8 | 48.6 |
|  | 2010 |  | 177 | 4,171 | 4.2 | 8.3 | 962 | 4,271 | 22.5 | 44.5 |
|  | 2011 |  | 194 | 4,503 | 4.3 | 8.5 | 1,031 | 4,612 | 22.4 | 44.3 |
|  | 2005 | BZDRs | 80 | 1,594 | 5 | 9.4 | 433 | 1,654 | 26.2 | 48.4 |
|  | 2006 |  | 204 | 3,203 | 6.4 | 13.2 | 905 | 3,308 | 27.4 | 56.7 |
|  | 2007 |  | 172 | 3,469 | 5 | 9.8 | 1,003 | 3,620 | 27.7 | 54.1 |
|  | 2008 |  | 181 | 3,888 | 4.7 | 9.2 | 1,082 | 4,038 | 26.8 | 52.6 |
|  | 2009 |  | 183 | 4,097 | 4.5 | 9.2 | 1,104 | 4,247 | 26 | 53.1 |
|  | 2010 |  | 161 | 4,102 | 3.9 | 7.8 | 1,047 | 4,271 | 24.5 | 48.2 |
|  | 2011 |  | 161 | 4,454 | 3.6 | 7.2 | 1,038 | 4,612 | 22.5 | 44.2 |
| Oulu | 2005 | Any | 104 | 1,204 | 8.6 | 16.5 | 600 | 1,342 | 44.7 | 80.1 |
|  | 2006 |  | 175 | 2,345 | 7.5 | 15.2 | 1,176 | 2,591 | 45.4 | 91.7 |
|  | 2007 |  | 176 | 2,304 | 7.6 | 16 | 1,181 | 2,547 | 46.4 | 97.2 |
|  | 2008 |  | 172 | 2,415 | 7.1 | 15.1 | 1,264 | 2,675 | 47.3 | 97.3 |
|  | 2009 |  | 174 | 2,660 | 6.5 | 13.2 | 1,432 | 2,974 | 48.2 | 96.5 |
|  | 2010 |  | 181 | 2,772 | 6.5 | 13.4 | 1,445 | 3,045 | 47.5 | 94.2 |
|  | 2011 |  | 171 | 2,749 | 6.2 | 12.7 | 1,377 | 3,054 | 45.1 | 90.1 |
|  | 2005 | Antipsychotics | 70 | 1,318 | 5.3 | 10 | 173 | 1,342 | 12.9 | 24 |
|  | 2006 |  | 118 | 2,559 | 4.6 | 9.4 | 351 | 2,591 | 13.5 | 27.8 |
|  | 2007 |  | 133 | 2,511 | 5.3 | 10.8 | 387 | 2,547 | 15.2 | 30.9 |
|  | 2008 |  | 153 | 2,621 | 5.8 | 12.2 | 439 | 2,675 | 16.4 | 33.6 |
|  | 2009 |  | 153 | 2,923 | 5.2 | 10.8 | 473 | 2,974 | 15.9 | 31.6 |
|  | 2010 |  | 143 | 2,999 | 4.8 | 9.5 | 471 | 3,045 | 15.5 | 30.5 |
|  | 2011 |  | 168 | 2,993 | 5.6 | 11.2 | 490 | 3,054 | 16 | 32.2 |
|  | 2005 | Antidepressants | 64 | 1,295 | 4.9 | 8.7 | 316 | 1,342 | 23.5 | 41.2 |
|  | 2006 |  | 91 | 2,540 | 3.6 | 7.3 | 579 | 2,591 | 22.3 | 44.7 |
|  | 2007 |  | 122 | 2,489 | 4.9 | 10 | 566 | 2,547 | 22.2 | 47.3 |
|  | 2008 |  | 100 | 2,608 | 3.8 | 8 | 598 | 2,675 | 22.4 | 45.5 |
|  | 2009 |  | 114 | 2,905 | 3.9 | 7.7 | 679 | 2,974 | 22.8 | 45.4 |
|  | 2010 |  | 113 | 2,972 | 3.8 | 7.7 | 713 | 3,045 | 23.4 | 46.4 |
|  | 2011 |  | 94 | 2,973 | 3.2 | 6.4 | 693 | 3,054 | 22.7 | 45.7 |
|  | 2005 | BZDRs | 78 | 1,293 | 6 | 11 | 376 | 1,342 | 28 | 50.4 |
|  | 2006 |  | 135 | 2,492 | 5.4 | 10.9 | 731 | 2,591 | 28.2 | 57.6 |
|  | 2007 |  | 98 | 2,451 | 4 | 8.2 | 712 | 2,547 | 28 | 60.1 |
|  | 2008 |  | 102 | 2,583 | 3.9 | 8.2 | 740 | 2,675 | 27.7 | 57.4 |
|  | 2009 |  | 123 | 2,861 | 4.3 | 8.6 | 862 | 2,974 | 29 | 58.7 |
|  | 2010 |  | 102 | 2,934 | 3.5 | 6.9 | 804 | 3,045 | 26.4 | 52.6 |
|  | 2011 |  | 92 | 2,943 | 3.1 | 6.3 | 731 | 3,054 | 23.9 | 47.7 |
| Turku | 2005 | Any | 177 | 1,710 | 10.4 | 19.5 | 952 | 1,942 | 49 | 91.6 |
|  | 2006 |  | 262 | 3,303 | 7.9 | 16.5 | 1,886 | 3,709 | 50.8 | 103.7 |
|  | 2007 |  | 285 | 3,464 | 8.2 | 16.7 | 2,013 | 3,912 | 51.5 | 99.7 |
|  | 2008 |  | 285 | 3,815 | 7.5 | 15.2 | 2,131 | 4,270 | 49.9 | 98.8 |
|  | 2009 |  | 301 | 3,874 | 7.8 | 16.1 | 2,076 | 4,283 | 48.5 | 97.1 |
|  | 2010 |  | 326 | 3,902 | 8.4 | 17.1 | 2,099 | 4,362 | 48.1 | 95.6 |
|  | 2011 |  | 324 | 4,335 | 7.5 | 15.1 | 2,292 | 4,820 | 47.6 | 93.8 |
|  | 2005 | Antipsychotics | 139 | 1,908 | 7.3 | 15 | 301 | 1,942 | 15.5 | 30.2 |
|  | 2006 |  | 188 | 3,651 | 5.1 | 10.5 | 585 | 3,709 | 15.8 | 32.2 |
|  | 2007 |  | 205 | 3,864 | 5.3 | 10.4 | 602 | 3,912 | 15.4 | 30 |
|  | 2008 |  | 235 | 4,216 | 5.6 | 11.1 | 649 | 4,270 | 15.2 | 30.1 |
|  | 2009 |  | 218 | 4,218 | 5.2 | 10.4 | 672 | 4,283 | 15.7 | 31.5 |
|  | 2010 |  | 271 | 4,286 | 6.3 | 12.6 | 725 | 4,362 | 16.6 | 33 |
|  | 2011 |  | 281 | 4,751 | 5.9 | 11.7 | 820 | 4,820 | 17 | 33.6 |
|  | 2005 | Antidepressants | 121 | 1,883 | 6.4 | 11.7 | 491 | 1,942 | 25.3 | 45.8 |
|  | 2006 |  | 188 | 3,598 | 5.2 | 10.7 | 930 | 3,709 | 25.1 | 50.8 |
|  | 2007 |  | 178 | 3,781 | 4.7 | 9.2 | 1,001 | 3,912 | 25.6 | 49.4 |
|  | 2008 |  | 200 | 4,127 | 4.8 | 9.6 | 1,107 | 4,270 | 25.9 | 51 |
|  | 2009 |  | 194 | 4,180 | 4.6 | 9.4 | 1,089 | 4,283 | 25.4 | 50.9 |
|  | 2010 |  | 197 | 4,232 | 4.7 | 9.3 | 1,090 | 4,362 | 25 | 49.8 |
|  | 2011 |  | 209 | 4,687 | 4.5 | 9 | 1,192 | 4,820 | 24.7 | 49.3 |
|  | 2005 | BZDRs | 122 | 1,865 | 6.5 | 13.8 | 604 | 1,942 | 31.1 | 59.1 |
|  | 2006 |  | 173 | 3,564 | 4.9 | 9.9 | 1,172 | 3,709 | 31.6 | 64.6 |
|  | 2007 |  | 183 | 3,717 | 4.9 | 9.9 | 1,227 | 3,912 | 31.4 | 60.9 |
|  | 2008 |  | 170 | 4,105 | 4.1 | 8.3 | 1,243 | 4,270 | 29.1 | 57.8 |
|  | 2009 |  | 170 | 4,145 | 4.1 | 8.3 | 1,142 | 4,283 | 26.7 | 53.4 |
|  | 2010 |  | 194 | 4,212 | 4.6 | 9.2 | 1,140 | 4,362 | 26.1 | 51.9 |
|  | 2011 |  | 196 | 4,666 | 4.2 | 8.4 | 1,202 | 4,820 | 24.9 | 49.1 |
| Tampere | 2005 | Any | 119 | 1,241 | 9.6 | 18.1 | 690 | 1,397 | 49.4 | 89.8 |
|  | 2006 |  | 190 | 2,509 | 7.6 | 15.8 | 1,368 | 2,804 | 48.8 | 99.4 |
|  | 2007 |  | 205 | 2,487 | 8.2 | 16.3 | 1,376 | 2,775 | 49.6 | 95 |
|  | 2008 |  | 205 | 2,563 | 8 | 16.4 | 1,412 | 2,835 | 49.8 | 99.7 |
|  | 2009 |  | 196 | 2,726 | 7.2 | 14.7 | 1,440 | 3,025 | 47.6 | 95.6 |
|  | 2010 |  | 219 | 2,705 | 8.1 | 16.4 | 1,383 | 2,963 | 46.7 | 92.3 |
|  | 2011 |  | 236 | 2,873 | 8.2 | 16.8 | 1,512 | 3,159 | 47.9 | 94.7 |
|  | 2005 | Antipsychotics | 110 | 1,373 | 8 | 15.1 | 224 | 1,397 | 16 | 29.6 |
|  | 2006 |  | 145 | 2,749 | 5.3 | 10.7 | 453 | 2,804 | 16.2 | 33.2 |
|  | 2007 |  | 168 | 2,735 | 6.1 | 11.9 | 452 | 2,775 | 16.3 | 31.2 |
|  | 2008 |  | 194 | 2,787 | 7 | 14 | 517 | 2,835 | 18.2 | 36.4 |
|  | 2009 |  | 173 | 2,978 | 5.8 | 11.5 | 513 | 3,025 | 17 | 33.9 |
|  | 2010 |  | 191 | 2,919 | 6.5 | 12.9 | 503 | 2,963 | 17 | 33.6 |
|  | 2011 |  | 169 | 3,099 | 5.5 | 10.9 | 554 | 3,159 | 17.5 | 34.9 |
|  | 2005 | Antidepressants | 72 | 1,357 | 5.3 | 9.5 | 368 | 1,397 | 26.3 | 47.8 |
|  | 2006 |  | 116 | 2,730 | 4.2 | 8.6 | 700 | 2,804 | 25 | 50.4 |
|  | 2007 |  | 132 | 2,694 | 4.9 | 9.6 | 702 | 2,775 | 25.3 | 48.7 |
|  | 2008 |  | 113 | 2,765 | 4.1 | 8.2 | 713 | 2,835 | 25.1 | 50.5 |
|  | 2009 |  | 106 | 2,947 | 3.6 | 7.3 | 717 | 3,025 | 23.7 | 47.9 |
|  | 2010 |  | 108 | 2,894 | 3.7 | 7.4 | 685 | 2,963 | 23.1 | 46.2 |
|  | 2011 |  | 149 | 3,094 | 4.8 | 9.7 | 768 | 3,159 | 24.3 | 48.8 |
|  | 2005 | BZDRs | 74 | 1,340 | 5.5 | 10.1 | 399 | 1,397 | 28.6 | 52.1 |
|  | 2006 |  | 122 | 2,697 | 4.5 | 9.4 | 829 | 2,804 | 29.6 | 60.3 |
|  | 2007 |  | 118 | 2,680 | 4.4 | 8.5 | 831 | 2,775 | 29.9 | 57 |
|  | 2008 |  | 123 | 2,745 | 4.5 | 9 | 776 | 2,835 | 27.4 | 54.6 |
|  | 2009 |  | 122 | 2,932 | 4.2 | 8.4 | 772 | 3,025 | 25.5 | 51 |
|  | 2010 |  | 108 | 2,861 | 3.8 | 7.6 | 716 | 2,963 | 24.2 | 47.6 |
|  | 2011 |  | 132 | 3,056 | 4.3 | 8.7 | 770 | 3,159 | 24.4 | 47.8 |
