# Supplementary material for: Regional changes in psychotropic use among Finnish persons with newly diagnosed Alzheimer’s disease in 2005-2011
Source: PLoS One. 2017 Mar 9;12(3):e0173450. doi: 10.1371/journal.pone.0173450 (PMC5344395; doi:10.1371/journal.pone.0173450)
Supplement: S2 Table — Rates are given as users/100 person-years. BZDRs Benzodiazepines and related drugs. Numbers in the hospital district correspond to those in Fig 2 (DOCX) [file pone.0173450.s002.docx]

S2 Table Incidence and prevalence of psychotropic use in hospital districts in 2005-2011.Rates are given as users/100 person-years. BZDRs Benzodiazepines and related drugs. Numbers in the hospital district correspond to those in Figure 1

| Hospital district | Year | Drug |  | Incidence | | | Prevalence | | | |
| --- | --- | --- | --- | --- | --- | --- | --- | --- | --- | --- |
|  |  |  | n of users | n of eligible persons for this analysis | Proportion of users (%) | Age- and sex-adjusted rate (users/ 100 person-years) | n of users | n of eligible persons for this analysis | Proportion of users (%) | Age- and sex-adjusted rate (users/ 100 person-years) |
| 1.Etelä-Karjala | 2005 | Any | 17 | 192 | 8.9 | 16.9 | 97 | 211 | 46.0 | 92.8 |
| Etelä-Karjala | 2006 |  | 31 | 352 | 8.8 | 16.3 | 194 | 394 | 49.2 | 89.6 |
| Etelä-Karjala | 2007 |  | 20 | 345 | 5.8 | 12.6 | 172 | 387 | 44.4 | 97.0 |
| Etelä-Karjala | 2008 |  | 24 | 395 | 6.1 | 12.4 | 176 | 436 | 40.4 | 82.8 |
| Etelä-Karjala | 2009 |  | 28 | 414 | 6.8 | 13.2 | 190 | 448 | 42.4 | 80.6 |
| Etelä-Karjala | 2010 |  | 36 | 459 | 7.8 | 16.0 | 209 | 510 | 41.0 | 82.2 |
| Etelä-Karjala | 2011 |  | 49 | 670 | 7.3 | 14.7 | 291 | 735 | 39.6 | 78.5 |
| Etelä-Karjala | 2005 | Antipsychotics | 10 | 206 | 4.9 | 8.7 | 31 | 211 | 14.7 | 28.5 |
| Etelä-Karjala | 2006 |  | 32 | 389 | 8.2 | 15.0 | 75 | 394 | 19.0 | 34.6 |
| Etelä-Karjala | 2007 |  | 24 | 379 | 6.3 | 14.3 | 74 | 387 | 19.1 | 40.9 |
| Etelä-Karjala | 2008 |  | 18 | 430 | 4.2 | 8.1 | 68 | 436 | 15.6 | 31.5 |
| Etelä-Karjala | 2009 |  | 20 | 446 | 4.5 | 8.1 | 54 | 448 | 12.1 | 22.0 |
| Etelä-Karjala | 2010 |  | 31 | 498 | 6.2 | 12.1 | 79 | 510 | 15.5 | 31.2 |
| Etelä-Karjala | 2011 |  | 37 | 722 | 5.1 | 10.0 | 105 | 735 | 14.3 | 28.6 |
| Etelä-Karjala | 2005 | Antidepressants | 12 | 208 | 5.8 | 12.5 | 45 | 211 | 21.3 | 44.3 |
| Etelä-Karjala | 2006 |  | 15 | 386 | 3.9 | 6.8 | 91 | 394 | 23.1 | 41.1 |
| Etelä-Karjala | 2007 |  | 11 | 379 | 2.9 | 5.9 | 82 | 387 | 21.2 | 45.5 |
| Etelä-Karjala | 2008 |  | 13 | 424 | 3.1 | 6.4 | 79 | 436 | 18.1 | 36.6 |
| Etelä-Karjala | 2009 |  | 14 | 439 | 3.2 | 6.0 | 93 | 448 | 20.8 | 39.9 |
| Etelä-Karjala | 2010 |  | 17 | 500 | 3.4 | 7.0 | 91 | 510 | 17.8 | 36.1 |
| Etelä-Karjala | 2011 |  | 32 | 715 | 4.5 | 8.7 | 146 | 735 | 19.9 | 40.0 |
| Etelä-Karjala | 2005 | BZDRs | 5 | 202 | 2.5 | 4.5 | 52 | 211 | 24.6 | 48.2 |
| Etelä-Karjala | 2006 |  | 17 | 380 | 4.5 | 7.7 | 99 | 394 | 25.1 | 46.0 |
| Etelä-Karjala | 2007 |  | 10 | 366 | 2.7 | 6.2 | 94 | 387 | 24.3 | 53.5 |
| Etelä-Karjala | 2008 |  | 19 | 423 | 4.5 | 9.4 | 101 | 436 | 23.2 | 47.5 |
| Etelä-Karjala | 2009 |  | 15 | 433 | 3.5 | 6.7 | 101 | 448 | 22.5 | 42.6 |
| Etelä-Karjala | 2010 |  | 22 | 487 | 4.5 | 9.3 | 111 | 510 | 21.8 | 43.1 |
| Etelä-Karjala | 2011 |  | 30 | 711 | 4.2 | 8.4 | 161 | 735 | 21.9 | 43.4 |
| 2.Etelä-Pohjanmaa | 2005 | Any | 40 | 432 | 9.3 | 17.2 | 243 | 492 | 49.4 | 93.2 |
| Etelä-Pohjanmaa | 2006 |  | 56 | 826 | 6.8 | 15.1 | 487 | 927 | 52.5 | 111.2 |
| Etelä-Pohjanmaa | 2007 |  | 67 | 894 | 7.5 | 14.9 | 504 | 998 | 50.5 | 94.7 |
| Etelä-Pohjanmaa | 2008 |  | 54 | 938 | 5.8 | 12.5 | 498 | 1,034 | 48.2 | 101.7 |
| Etelä-Pohjanmaa | 2009 |  | 56 | 877 | 6.4 | 13.5 | 466 | 978 | 47.6 | 95.9 |
| Etelä-Pohjanmaa | 2010 |  | 66 | 918 | 7.2 | 14.3 | 485 | 1,033 | 47.0 | 94.5 |
| Etelä-Pohjanmaa | 2011 |  | 62 | 1,054 | 5.9 | 12.1 | 524 | 1,158 | 45.3 | 90.4 |
| Etelä-Pohjanmaa | 2005 | Antipsychotics | 32 | 483 | 6.6 | 12.1 | 79 | 492 | 16.1 | 29.9 |
| Etelä-Pohjanmaa | 2006 |  | 41 | 913 | 4.5 | 9.6 | 142 | 927 | 15.3 | 32.2 |
| Etelä-Pohjanmaa | 2007 |  | 40 | 982 | 4.1 | 7.6 | 142 | 998 | 14.2 | 27.0 |
| Etelä-Pohjanmaa | 2008 |  | 49 | 1,018 | 4.8 | 10.0 | 152 | 1,034 | 14.7 | 30.7 |
| Etelä-Pohjanmaa | 2009 |  | 39 | 952 | 4.1 | 8.4 | 165 | 978 | 16.9 | 33.9 |
| Etelä-Pohjanmaa | 2010 |  | 58 | 1,010 | 5.7 | 11.4 | 177 | 1,033 | 17.1 | 34.4 |
| Etelä-Pohjanmaa | 2011 |  | 41 | 1,137 | 3.6 | 7.4 | 177 | 1,158 | 15.3 | 30.3 |
| Etelä-Pohjanmaa | 2005 | Antidepressants | 33 | 481 | 6.9 | 12.7 | 121 | 492 | 24.6 | 46.5 |
| Etelä-Pohjanmaa | 2006 |  | 39 | 912 | 4.3 | 8.9 | 228 | 927 | 24.6 | 52.1 |
| Etelä-Pohjanmaa | 2007 |  | 50 | 972 | 5.1 | 9.8 | 241 | 998 | 24.1 | 45.5 |
| Etelä-Pohjanmaa | 2008 |  | 40 | 1,001 | 4.0 | 8.4 | 244 | 1,034 | 23.6 | 49.4 |
| Etelä-Pohjanmaa | 2009 |  | 30 | 953 | 3.1 | 6.5 | 231 | 978 | 23.6 | 47.8 |
| Etelä-Pohjanmaa | 2010 |  | 44 | 1,006 | 4.4 | 8.8 | 241 | 1,033 | 23.3 | 47.6 |
| Etelä-Pohjanmaa | 2011 |  | 47 | 1,134 | 4.1 | 8.5 | 255 | 1,158 | 22.0 | 45.0 |
| Etelä-Pohjanmaa | 2005 | BZDRs | 31 | 469 | 6.6 | 13.0 | 164 | 492 | 33.3 | 63.4 |
| Etelä-Pohjanmaa | 2006 |  | 44 | 888 | 5.0 | 10.7 | 311 | 927 | 33.5 | 71.2 |
| Etelä-Pohjanmaa | 2007 |  | 42 | 955 | 4.4 | 8.8 | 313 | 998 | 31.4 | 58.6 |
| Etelä-Pohjanmaa | 2008 |  | 32 | 1,001 | 3.2 | 6.7 | 288 | 1,034 | 27.9 | 58.7 |
| Etelä-Pohjanmaa | 2009 |  | 44 | 948 | 4.6 | 9.7 | 265 | 978 | 27.1 | 54.5 |
| Etelä-Pohjanmaa | 2010 |  | 49 | 994 | 4.9 | 10.1 | 274 | 1,033 | 26.5 | 53.8 |
| Etelä-Pohjanmaa | 2011 |  | 40 | 1,123 | 3.6 | 7.2 | 279 | 1,158 | 24.1 | 48.3 |
| 3.Etelä-Savo | 2005 | Any | 26 | 260 | 10.0 | 17.6 | 141 | 294 | 48.0 | 83.8 |
| Etelä-Savo | 2006 |  | 40 | 480 | 8.3 | 17.6 | 245 | 531 | 46.1 | 100.1 |
| Etelä-Savo | 2007 |  | 38 | 498 | 7.6 | 14.9 | 264 | 559 | 47.2 | 91.8 |
| Etelä-Savo | 2008 |  | 48 | 522 | 9.2 | 18.7 | 278 | 579 | 48.0 | 94.5 |
| Etelä-Savo | 2009 |  | 33 | 594 | 5.6 | 11.3 | 290 | 651 | 44.5 | 90.6 |
| Etelä-Savo | 2010 |  | 48 | 692 | 6.9 | 14.0 | 342 | 760 | 45.0 | 89.9 |
| Etelä-Savo | 2011 |  | 52 | 763 | 6.8 | 13.1 | 394 | 840 | 46.9 | 90.8 |
| Etelä-Savo | 2005 | Antipsychotics | 24 | 287 | 8.4 | 13.9 | 61 | 294 | 20.7 | 36.5 |
| Etelä-Savo | 2006 |  | 34 | 519 | 6.6 | 14.1 | 117 | 531 | 22.0 | 48.6 |
| Etelä-Savo | 2007 |  | 32 | 550 | 5.8 | 11.7 | 103 | 559 | 18.4 | 36.6 |
| Etelä-Savo | 2008 |  | 35 | 571 | 6.1 | 12.1 | 104 | 579 | 18.0 | 35.2 |
| Etelä-Savo | 2009 |  | 35 | 646 | 5.4 | 11.0 | 116 | 651 | 17.8 | 36.1 |
| Etelä-Savo | 2010 |  | 33 | 746 | 4.4 | 8.4 | 117 | 760 | 15.4 | 30.4 |
| Etelä-Savo | 2011 |  | 38 | 828 | 4.6 | 8.9 | 127 | 840 | 15.1 | 29.8 |
| Etelä-Savo | 2005 | Antidepressants | 12 | 290 | 4.1 | 7.0 | 58 | 294 | 19.7 | 35.0 |
| Etelä-Savo | 2006 |  | 21 | 520 | 4.0 | 8.0 | 97 | 531 | 18.3 | 39.7 |
| Etelä-Savo | 2007 |  | 20 | 552 | 3.6 | 6.7 | 106 | 559 | 19.0 | 36.5 |
| Etelä-Savo | 2008 |  | 21 | 571 | 3.7 | 7.2 | 115 | 579 | 19.9 | 39.0 |
| Etelä-Savo | 2009 |  | 22 | 640 | 3.4 | 7.1 | 128 | 651 | 19.7 | 41.0 |
| Etelä-Savo | 2010 |  | 34 | 750 | 4.5 | 9.3 | 162 | 760 | 21.3 | 43.8 |
| Etelä-Savo | 2011 |  | 38 | 820 | 4.6 | 8.7 | 186 | 840 | 22.1 | 43.0 |
| Etelä-Savo | 2005 | BZDRs | 7 | 280 | 2.5 | 4.3 | 85 | 294 | 28.9 | 50.8 |
| Etelä-Savo | 2006 |  | 25 | 511 | 4.9 | 10.9 | 142 | 531 | 26.7 | 58.0 |
| Etelä-Savo | 2007 |  | 18 | 524 | 3.4 | 6.7 | 160 | 559 | 28.6 | 55.5 |
| Etelä-Savo | 2008 |  | 29 | 548 | 5.3 | 10.6 | 172 | 579 | 29.7 | 58.4 |
| Etelä-Savo | 2009 |  | 28 | 631 | 4.4 | 9.0 | 173 | 651 | 26.6 | 54.0 |
| Etelä-Savo | 2010 |  | 35 | 731 | 4.8 | 9.3 | 201 | 760 | 26.4 | 52.9 |
| Etelä-Savo | 2011 |  | 31 | 812 | 3.8 | 7.3 | 223 | 840 | 26.5 | 50.5 |
| 4.Helsinki ja Uusimaa | 2005 | Any | 162 | 1,292 | 12.5 | 24.2 | 797 | 1,507 | 52.9 | 97.4 |
| Helsinki ja Uusimaa | 2006 |  | 250 | 2,690 | 9.3 | 19.1 | 1650 | 3,091 | 53.4 | 106.1 |
| Helsinki ja Uusimaa | 2007 |  | 235 | 3,020 | 7.8 | 16.1 | 1769 | 3,444 | 51.4 | 101.5 |
| Helsinki ja Uusimaa | 2008 |  | 347 | 3,336 | 10.4 | 20.6 | 2028 | 3,813 | 53.2 | 102.0 |
| Helsinki ja Uusimaa | 2009 |  | 296 | 3,528 | 8.4 | 17.7 | 2152 | 4,075 | 52.8 | 107.7 |
| Helsinki ja Uusimaa | 2010 |  | 299 | 3,685 | 8.1 | 16.8 | 2130 | 4,228 | 50.4 | 100.0 |
| Helsinki ja Uusimaa | 2011 |  | 343 | 4,092 | 8.4 | 16.8 | 2213 | 4,649 | 47.6 | 91.4 |
| Helsinki ja Uusimaa | 2005 | Antipsychotics | 124 | 1,483 | 8.4 | 15.6 | 247 | 1,507 | 16.4 | 30.1 |
| Helsinki ja Uusimaa | 2006 |  | 181 | 3,035 | 6.0 | 11.9 | 516 | 3,091 | 16.7 | 33.0 |
| Helsinki ja Uusimaa | 2007 |  | 163 | 3,391 | 4.8 | 9.6 | 493 | 3,444 | 14.3 | 28.2 |
| Helsinki ja Uusimaa | 2008 |  | 281 | 3,740 | 7.5 | 14.5 | 671 | 3,813 | 17.6 | 33.5 |
| Helsinki ja Uusimaa | 2009 |  | 262 | 3,988 | 6.6 | 13.4 | 747 | 4,075 | 18.3 | 37.1 |
| Helsinki ja Uusimaa | 2010 |  | 238 | 4,152 | 5.7 | 11.6 | 707 | 4,228 | 16.7 | 33.1 |
| Helsinki ja Uusimaa | 2011 |  | 274 | 4,563 | 6.0 | 11.7 | 721 | 4,649 | 15.5 | 29.7 |
| Helsinki ja Uusimaa | 2005 | Antidepressants | 123 | 1,441 | 8.5 | 15.7 | 489 | 1,507 | 32.4 | 59.5 |
| Helsinki ja Uusimaa | 2006 |  | 186 | 2,971 | 6.3 | 12.5 | 1011 | 3,091 | 32.7 | 64.6 |
| Helsinki ja Uusimaa | 2007 |  | 189 | 3,312 | 5.7 | 11.3 | 1119 | 3,444 | 32.5 | 63.8 |
| Helsinki ja Uusimaa | 2008 |  | 235 | 3,676 | 6.4 | 12.3 | 1247 | 3,813 | 32.7 | 62.3 |
| Helsinki ja Uusimaa | 2009 |  | 246 | 3,917 | 6.3 | 12.9 | 1318 | 4,075 | 32.3 | 65.8 |
| Helsinki ja Uusimaa | 2010 |  | 211 | 4,030 | 5.2 | 10.5 | 1314 | 4,228 | 31.1 | 61.4 |
| Helsinki ja Uusimaa | 2011 |  | 254 | 4,461 | 5.7 | 11.1 | 1368 | 4,649 | 29.4 | 56.2 |
| Helsinki ja Uusimaa | 2005 | BZDRs | 114 | 1,425 | 8.0 | 15.2 | 428 | 1,507 | 28.4 | 52.5 |
| Helsinki ja Uusimaa | 2006 |  | 161 | 2,951 | 5.5 | 11.0 | 889 | 3,091 | 28.8 | 57.5 |
| Helsinki ja Uusimaa | 2007 |  | 180 | 3,282 | 5.5 | 11.0 | 964 | 3,444 | 28.0 | 55.6 |
| Helsinki ja Uusimaa | 2008 |  | 211 | 3,667 | 5.8 | 11.2 | 1022 | 3,813 | 26.8 | 51.8 |
| Helsinki ja Uusimaa | 2009 |  | 174 | 3,889 | 4.5 | 9.3 | 1045 | 4,075 | 25.6 | 52.6 |
| Helsinki ja Uusimaa | 2010 |  | 191 | 4,042 | 4.7 | 9.5 | 1054 | 4,228 | 24.9 | 49.9 |
| Helsinki ja Uusimaa | 2011 |  | 184 | 4,475 | 4.1 | 8.0 | 1048 | 4,649 | 22.5 | 43.6 |
| 5.Itä-Savo | 2005 | Any | 8 | 128 | 6.3 | 12.3 | 53 | 140 | 37.9 | 74.6 |
| Itä-Savo | 2006 |  | 23 | 244 | 9.4 | 18.9 | 110 | 265 | 41.5 | 86.7 |
| Itä-Savo | 2007 |  | 18 | 240 | 7.5 | 17.4 | 117 | 261 | 44.8 | 97.8 |
| Itä-Savo | 2008 |  | 23 | 261 | 8.8 | 16.9 | 133 | 285 | 46.7 | 89.9 |
| Itä-Savo | 2009 |  | 27 | 301 | 9.0 | 19.3 | 153 | 330 | 46.4 | 92.2 |
| Itä-Savo | 2010 |  | 15 | 301 | 5.0 | 10.3 | 137 | 325 | 42.2 | 86.2 |
| Itä-Savo | 2011 |  | 22 | 275 | 8.0 | 14.7 | 113 | 291 | 38.8 | 76.0 |
| Itä-Savo | 2005 | Antipsychotics | 9 | 139 | 6.5 | 12.2 | 25 | 140 | 17.9 | 34.9 |
| Itä-Savo | 2006 |  | 13 | 259 | 5.0 | 9.7 | 45 | 265 | 17.0 | 34.9 |
| Itä-Savo | 2007 |  | 19 | 257 | 7.4 | 17.7 | 44 | 261 | 16.9 | 37.3 |
| Itä-Savo | 2008 |  | 21 | 282 | 7.4 | 13.4 | 46 | 285 | 16.1 | 31.3 |
| Itä-Savo | 2009 |  | 28 | 326 | 8.6 | 16.7 | 57 | 330 | 17.3 | 36.2 |
| Itä-Savo | 2010 |  | 16 | 323 | 5.0 | 9.9 | 47 | 325 | 14.5 | 29.5 |
| Itä-Savo | 2011 |  | 15 | 291 | 5.2 | 9.4 | 32 | 291 | 11.0 | 20.2 |
| Itä-Savo | 2005 | Antidepressants | 3 | 138 | 2.2 | 4.6 | 26 | 140 | 18.6 | 37.2 |
| Itä-Savo | 2006 |  | 9 | 263 | 3.4 | 8.0 | 51 | 265 | 19.2 | 42.6 |
| Itä-Savo | 2007 |  | 11 | 258 | 4.3 | 11.4 | 64 | 261 | 24.5 | 56.5 |
| Itä-Savo | 2008 |  | 9 | 279 | 3.2 | 6.2 | 72 | 285 | 25.3 | 49.5 |
| Itä-Savo | 2009 |  | 13 | 324 | 4.0 | 8.7 | 81 | 330 | 24.5 | 48.4 |
| Itä-Savo | 2010 |  | 7 | 319 | 2.2 | 4.6 | 63 | 325 | 19.4 | 40.2 |
| Itä-Savo | 2011 |  | 12 | 287 | 4.2 | 7.7 | 54 | 291 | 18.6 | 37.2 |
| Itä-Savo | 2005 | BZDRs | 2 | 136 | 1.5 | 2.6 | 18 | 140 | 12.9 | 25.3 |
| Itä-Savo | 2006 |  | 19 | 253 | 7.5 | 14.9 | 59 | 265 | 22.3 | 46.2 |
| Itä-Savo | 2007 |  | 10 | 254 | 3.9 | 7.8 | 62 | 261 | 23.8 | 51.2 |
| Itä-Savo | 2008 |  | 8 | 277 | 2.9 | 5.7 | 62 | 285 | 21.8 | 40.3 |
| Itä-Savo | 2009 |  | 15 | 323 | 4.6 | 9.3 | 71 | 330 | 21.5 | 42.0 |
| Itä-Savo | 2010 |  | 8 | 317 | 2.5 | 5.3 | 59 | 325 | 18.2 | 37.5 |
| Itä-Savo | 2011 |  | 8 | 285 | 2.8 | 5.0 | 48 | 291 | 16.5 | 30.9 |
| 6.Kainuu | 2005 | Any | 10 | 164 | 6.1 | 10.3 | 53 | 168 | 31.5 | 59.6 |
| Kainuu | 2006 |  | 28 | 314 | 8.9 | 17.0 | 132 | 344 | 38.4 | 73.0 |
| Kainuu | 2007 |  | 22 | 327 | 6.7 | 15.1 | 153 | 363 | 42.1 | 92.3 |
| Kainuu | 2008 |  | 18 | 334 | 5.4 | 11.2 | 155 | 363 | 42.7 | 91.2 |
| Kainuu | 2009 |  | 29 | 373 | 7.8 | 16.5 | 185 | 410 | 45.1 | 94.1 |
| Kainuu | 2010 |  | 24 | 382 | 6.3 | 12.0 | 181 | 424 | 42.7 | 84.7 |
| Kainuu | 2011 |  | 22 | 379 | 5.8 | 11.6 | 162 | 421 | 38.5 | 75.0 |
| Kainuu | 2005 | Antipsychotics | 4 | 168 | 2.4 | 3.7 | 13 | 168 | 7.7 | 16.3 |
| Kainuu | 2006 |  | 15 | 339 | 4.4 | 8.5 | 39 | 344 | 11.3 | 22.8 |
| Kainuu | 2007 |  | 23 | 355 | 6.5 | 13.2 | 64 | 363 | 17.6 | 38.2 |
| Kainuu | 2008 |  | 18 | 360 | 5.0 | 10.5 | 59 | 363 | 16.3 | 34.8 |
| Kainuu | 2009 |  | 26 | 405 | 6.4 | 14.2 | 60 | 410 | 14.6 | 31.1 |
| Kainuu | 2010 |  | 24 | 418 | 5.7 | 10.7 | 63 | 424 | 14.9 | 28.9 |
| Kainuu | 2011 |  | 24 | 411 | 5.8 | 10.7 | 66 | 421 | 15.7 | 30.9 |
| Kainuu | 2005 | Antidepressants | 6 | 166 | 3.6 | 5.9 | 27 | 168 | 16.1 | 30.7 |
| Kainuu | 2006 |  | 10 | 339 | 2.9 | 5.5 | 57 | 344 | 16.6 | 31.9 |
| Kainuu | 2007 |  | 15 | 360 | 4.2 | 9.8 | 63 | 363 | 17.4 | 37.8 |
| Kainuu | 2008 |  | 7 | 356 | 2.0 | 3.8 | 63 | 363 | 17.4 | 36.4 |
| Kainuu | 2009 |  | 13 | 403 | 3.2 | 6.3 | 87 | 410 | 21.2 | 43.4 |
| Kainuu | 2010 |  | 18 | 416 | 4.3 | 8.4 | 87 | 424 | 20.5 | 42.4 |
| Kainuu | 2011 |  | 8 | 411 | 1.9 | 3.7 | 71 | 421 | 16.9 | 33.5 |
| Kainuu | 2005 | BZDRs | 2 | 166 | 1.2 | 2.0 | 29 | 168 | 17.3 | 31.1 |
| Kainuu | 2006 |  | 23 | 335 | 6.9 | 12.9 | 83 | 344 | 24.1 | 44.2 |
| Kainuu | 2007 |  | 12 | 349 | 3.4 | 6.4 | 83 | 363 | 22.9 | 48.4 |
| Kainuu | 2008 |  | 13 | 353 | 3.7 | 7.8 | 85 | 363 | 23.4 | 50.7 |
| Kainuu | 2009 |  | 19 | 395 | 4.8 | 10.2 | 109 | 410 | 26.6 | 56.2 |
| Kainuu | 2010 |  | 17 | 409 | 4.2 | 7.6 | 102 | 424 | 24.1 | 46.1 |
| Kainuu | 2011 |  | 9 | 403 | 2.2 | 4.7 | 94 | 421 | 22.3 | 43.9 |
| 7.Kanta-Häme | 2005 | Any | 41 | 245 | 16.7 | 30.7 | 148 | 278 | 53.2 | 96.8 |
| Kanta-Häme | 2006 |  | 34 | 464 | 7.3 | 15.9 | 291 | 535 | 54.4 | 113.6 |
| Kanta-Häme | 2007 |  | 40 | 459 | 8.7 | 17.5 | 300 | 533 | 56.3 | 108.9 |
| Kanta-Häme | 2008 |  | 44 | 541 | 8.1 | 15.4 | 312 | 621 | 50.2 | 92.2 |
| Kanta-Häme | 2009 |  | 48 | 650 | 7.4 | 15.6 | 333 | 699 | 47.6 | 99.4 |
| Kanta-Häme | 2010 |  | 44 | 565 | 7.8 | 14.8 | 293 | 628 | 46.7 | 86.1 |
| Kanta-Häme | 2011 |  | 35 | 599 | 5.8 | 13.1 | 288 | 652 | 44.2 | 94.6 |
| Kanta-Häme | 2005 | Antipsychotics | 27 | 274 | 9.9 | 18.9 | 47 | 278 | 16.9 | 31.5 |
| Kanta-Häme | 2006 |  | 28 | 524 | 5.3 | 11.3 | 96 | 535 | 17.9 | 37.8 |
| Kanta-Häme | 2007 |  | 27 | 526 | 5.1 | 10.1 | 82 | 533 | 15.4 | 29.7 |
| Kanta-Häme | 2008 |  | 37 | 612 | 6.0 | 11.2 | 93 | 621 | 15.0 | 27.5 |
| Kanta-Häme | 2009 |  | 30 | 692 | 4.3 | 9.3 | 99 | 699 | 14.2 | 30.0 |
| Kanta-Häme | 2010 |  | 33 | 618 | 5.3 | 10.2 | 102 | 628 | 16.2 | 30.5 |
| Kanta-Häme | 2011 |  | 33 | 640 | 5.2 | 11.2 | 101 | 652 | 15.5 | 33.2 |
| Kanta-Häme | 2005 | Antidepressants | 28 | 272 | 10.3 | 18.5 | 90 | 278 | 32.4 | 58.0 |
| Kanta-Häme | 2006 |  | 24 | 507 | 4.7 | 10.7 | 160 | 535 | 29.9 | 62.5 |
| Kanta-Häme | 2007 |  | 28 | 502 | 5.6 | 11.0 | 170 | 533 | 31.9 | 61.1 |
| Kanta-Häme | 2008 |  | 32 | 594 | 5.4 | 9.9 | 166 | 621 | 26.7 | 49.0 |
| Kanta-Häme | 2009 |  | 29 | 689 | 4.2 | 8.5 | 173 | 699 | 24.7 | 51.1 |
| Kanta-Häme | 2010 |  | 31 | 611 | 5.1 | 9.4 | 165 | 628 | 26.3 | 48.2 |
| Kanta-Häme | 2011 |  | 20 | 637 | 3.1 | 6.8 | 151 | 652 | 23.2 | 50.0 |
| Kanta-Häme | 2005 | BZDRs | 23 | 271 | 8.5 | 16.9 | 81 | 278 | 29.1 | 53.9 |
| Kanta-Häme | 2006 |  | 28 | 510 | 5.5 | 11.1 | 169 | 535 | 31.6 | 64.4 |
| Kanta-Häme | 2007 |  | 25 | 504 | 5.0 | 9.6 | 168 | 533 | 31.5 | 60.5 |
| Kanta-Häme | 2008 |  | 31 | 590 | 5.3 | 9.7 | 196 | 621 | 31.6 | 57.7 |
| Kanta-Häme | 2009 |  | 20 | 686 | 2.9 | 6.1 | 182 | 699 | 26.0 | 54.8 |
| Kanta-Häme | 2010 |  | 23 | 605 | 3.8 | 7.2 | 152 | 628 | 24.2 | 44.6 |
| Kanta-Häme | 2011 |  | 14 | 639 | 2.2 | 5.0 | 142 | 652 | 21.8 | 46.1 |
| 8.Keski-Pohjanmaa | 2005 | Any | 10 | 115 | 8.7 | 16.2 | 67 | 129 | 51.9 | 91.1 |
| Keski-Pohjanmaa | 2006 |  | 15 | 217 | 6.9 | 12.2 | 118 | 240 | 49.2 | 98.4 |
| Keski-Pohjanmaa | 2007 |  | 19 | 244 | 7.8 | 20.5 | 146 | 272 | 53.7 | 114.7 |
| Keski-Pohjanmaa | 2008 |  | 22 | 296 | 7.4 | 16.3 | 169 | 331 | 51.1 | 110.2 |
| Keski-Pohjanmaa | 2009 |  | 15 | 324 | 4.6 | 9.9 | 186 | 362 | 51.4 | 111.0 |
| Keski-Pohjanmaa | 2010 |  | 21 | 333 | 6.3 | 13.8 | 196 | 372 | 52.7 | 105.4 |
| Keski-Pohjanmaa | 2011 |  | 12 | 317 | 3.8 | 7.6 | 165 | 355 | 46.5 | 89.0 |
| Keski-Pohjanmaa | 2005 | Antipsychotics | 8 | 123 | 6.5 | 12.0 | 22 | 129 | 17.1 | 30.5 |
| Keski-Pohjanmaa | 2006 |  | 14 | 235 | 6.0 | 11.1 | 41 | 240 | 17.1 | 35.8 |
| Keski-Pohjanmaa | 2007 |  | 16 | 265 | 6.0 | 16.9 | 57 | 272 | 21.0 | 45.1 |
| Keski-Pohjanmaa | 2008 |  | 18 | 323 | 5.6 | 10.5 | 63 | 331 | 19.0 | 47.5 |
| Keski-Pohjanmaa | 2009 |  | 12 | 353 | 3.4 | 7.4 | 57 | 362 | 15.7 | 34.1 |
| Keski-Pohjanmaa | 2010 |  | 21 | 369 | 5.7 | 11.1 | 69 | 372 | 18.5 | 35.9 |
| Keski-Pohjanmaa | 2011 |  | 12 | 351 | 3.4 | 6.7 | 54 | 355 | 15.2 | 28.6 |
| Keski-Pohjanmaa | 2005 | Antidepressants | 6 | 124 | 4.8 | 8.3 | 34 | 129 | 26.4 | 44.8 |
| Keski-Pohjanmaa | 2006 |  | 8 | 237 | 3.4 | 6.5 | 56 | 240 | 23.3 | 44.4 |
| Keski-Pohjanmaa | 2007 |  | 14 | 266 | 5.3 | 11.9 | 73 | 272 | 26.8 | 57.2 |
| Keski-Pohjanmaa | 2008 |  | 11 | 321 | 3.4 | 8.0 | 89 | 331 | 26.9 | 62.6 |
| Keski-Pohjanmaa | 2009 |  | 16 | 351 | 4.6 | 9.0 | 100 | 362 | 27.6 | 60.1 |
| Keski-Pohjanmaa | 2010 |  | 11 | 361 | 3.0 | 6.1 | 98 | 372 | 26.3 | 53.3 |
| Keski-Pohjanmaa | 2011 |  | 11 | 341 | 3.2 | 6.3 | 90 | 355 | 25.4 | 49.0 |
| Keski-Pohjanmaa | 2005 | BZDRs | 9 | 125 | 7.2 | 12.7 | 41 | 129 | 31.8 | 57.3 |
| Keski-Pohjanmaa | 2006 |  | 15 | 234 | 6.4 | 12.5 | 72 | 240 | 30.0 | 62.4 |
| Keski-Pohjanmaa | 2007 |  | 5 | 260 | 1.9 | 3.8 | 88 | 272 | 32.4 | 70.2 |
| Keski-Pohjanmaa | 2008 |  | 13 | 316 | 4.1 | 8.7 | 102 | 331 | 30.8 | 70.7 |
| Keski-Pohjanmaa | 2009 |  | 19 | 352 | 5.4 | 11.4 | 120 | 362 | 33.1 | 70.3 |
| Keski-Pohjanmaa | 2010 |  | 13 | 356 | 3.7 | 7.6 | 110 | 372 | 29.6 | 58.1 |
| Keski-Pohjanmaa | 2011 |  | 9 | 342 | 2.6 | 4.8 | 86 | 355 | 24.2 | 45.6 |
| 9.Keski-Suomi | 2005 | Any | 48 | 430 | 11.2 | 21.2 | 234 | 478 | 49.0 | 89.4 |
| Keski-Suomi | 2006 |  | 58 | 848 | 6.8 | 15.3 | 442 | 931 | 47.5 | 103.0 |
| Keski-Suomi | 2007 |  | 87 | 929 | 9.4 | 17.2 | 497 | 1,039 | 47.8 | 88.9 |
| Keski-Suomi | 2008 |  | 85 | 1,041 | 8.2 | 17.0 | 604 | 1,199 | 50.4 | 98.4 |
| Keski-Suomi | 2009 |  | 87 | 1,118 | 7.8 | 16.1 | 608 | 1,238 | 49.1 | 97.9 |
| Keski-Suomi | 2010 |  | 83 | 1,139 | 7.3 | 14.8 | 606 | 1,268 | 47.8 | 95.2 |
| Keski-Suomi | 2011 |  | 81 | 1,210 | 6.7 | 13.7 | 590 | 1,335 | 44.2 | 87.7 |
| Keski-Suomi | 2005 | Antipsychotics | 30 | 471 | 6.4 | 12.2 | 80 | 478 | 16.7 | 30.0 |
| Keski-Suomi | 2006 |  | 52 | 918 | 5.7 | 12.0 | 145 | 931 | 15.6 | 33.2 |
| Keski-Suomi | 2007 |  | 56 | 1,020 | 5.5 | 10.4 | 166 | 1,039 | 16.0 | 29.6 |
| Keski-Suomi | 2008 |  | 95 | 1,173 | 8.1 | 16.0 | 237 | 1,199 | 19.8 | 38.6 |
| Keski-Suomi | 2009 |  | 61 | 1,216 | 5.0 | 10.0 | 210 | 1,238 | 17.0 | 33.8 |
| Keski-Suomi | 2010 |  | 89 | 1,248 | 7.1 | 14.2 | 223 | 1,268 | 17.6 | 34.7 |
| Keski-Suomi | 2011 |  | 75 | 1,323 | 5.7 | 11.3 | 220 | 1,335 | 16.5 | 32.0 |
| Keski-Suomi | 2005 | Antidepressants | 32 | 465 | 6.9 | 12.2 | 110 | 478 | 23.0 | 41.5 |
| Keski-Suomi | 2006 |  | 31 | 911 | 3.4 | 7.2 | 201 | 931 | 21.6 | 46.1 |
| Keski-Suomi | 2007 |  | 49 | 1,009 | 4.9 | 8.8 | 238 | 1,039 | 22.9 | 42.2 |
| Keski-Suomi | 2008 |  | 68 | 1,155 | 5.9 | 11.8 | 298 | 1,199 | 24.9 | 48.1 |
| Keski-Suomi | 2009 |  | 49 | 1,210 | 4.0 | 8.1 | 307 | 1,238 | 24.8 | 49.4 |
| Keski-Suomi | 2010 |  | 44 | 1,236 | 3.6 | 7.1 | 282 | 1,268 | 22.2 | 44.0 |
| Keski-Suomi | 2011 |  | 48 | 1,298 | 3.7 | 7.4 | 280 | 1,335 | 21.0 | 41.6 |
| Keski-Suomi | 2005 | BZDRs | 32 | 461 | 6.9 | 12.4 | 146 | 478 | 30.5 | 55.3 |
| Keski-Suomi | 2006 |  | 46 | 905 | 5.1 | 11.3 | 279 | 931 | 30.0 | 65.1 |
| Keski-Suomi | 2007 |  | 56 | 1,001 | 5.6 | 10.7 | 309 | 1,039 | 29.7 | 55.9 |
| Keski-Suomi | 2008 |  | 59 | 1,148 | 5.1 | 10.4 | 366 | 1,199 | 30.5 | 59.5 |
| Keski-Suomi | 2009 |  | 55 | 1,189 | 4.6 | 9.5 | 355 | 1,238 | 28.7 | 57.2 |
| Keski-Suomi | 2010 |  | 46 | 1,213 | 3.8 | 7.7 | 353 | 1,268 | 27.8 | 55.7 |
| Keski-Suomi | 2011 |  | 50 | 1,287 | 3.9 | 7.8 | 314 | 1,335 | 23.5 | 46.6 |
| 10.Kymenlaakso | 2005 | Any | 44 | 357 | 12.3 | 24.1 | 173 | 386 | 44.8 | 81.1 |
| Kymenlaakso | 2006 |  | 49 | 720 | 6.8 | 14.5 | 335 | 787 | 42.6 | 88.9 |
| Kymenlaakso | 2007 |  | 61 | 802 | 7.6 | 15.3 | 393 | 862 | 45.6 | 92.6 |
| Kymenlaakso | 2008 |  | 79 | 847 | 9.3 | 18.4 | 418 | 924 | 45.2 | 86.3 |
| Kymenlaakso | 2009 |  | 56 | 873 | 6.4 | 13.6 | 422 | 962 | 43.9 | 92.1 |
| Kymenlaakso | 2010 |  | 87 | 1,010 | 8.6 | 16.9 | 529 | 1,109 | 47.7 | 93.9 |
| Kymenlaakso | 2011 |  | 71 | 1,114 | 6.4 | 13.1 | 560 | 1,230 | 45.5 | 94.2 |
| Kymenlaakso | 2005 | Antipsychotics | 30 | 383 | 7.8 | 15.2 | 63 | 386 | 16.3 | 29.9 |
| Kymenlaakso | 2006 |  | 42 | 782 | 5.4 | 11.2 | 109 | 787 | 13.9 | 29.2 |
| Kymenlaakso | 2007 |  | 35 | 858 | 4.1 | 8.3 | 117 | 862 | 13.6 | 26.9 |
| Kymenlaakso | 2008 |  | 63 | 912 | 6.9 | 13.4 | 141 | 924 | 15.3 | 28.9 |
| Kymenlaakso | 2009 |  | 43 | 951 | 4.5 | 9.5 | 149 | 962 | 15.5 | 32.4 |
| Kymenlaakso | 2010 |  | 74 | 1,099 | 6.7 | 13.3 | 192 | 1,109 | 17.3 | 34.4 |
| Kymenlaakso | 2011 |  | 73 | 1,211 | 6.0 | 12.3 | 217 | 1,230 | 17.6 | 37.3 |
| Kymenlaakso | 2005 | Antidepressants | 18 | 382 | 4.7 | 9.7 | 61 | 386 | 15.8 | 28.9 |
| Kymenlaakso | 2006 |  | 28 | 770 | 3.6 | 7.5 | 146 | 787 | 18.6 | 37.4 |
| Kymenlaakso | 2007 |  | 36 | 848 | 4.2 | 8.6 | 171 | 862 | 19.8 | 39.4 |
| Kymenlaakso | 2008 |  | 31 | 910 | 3.4 | 6.6 | 157 | 924 | 17.0 | 32.6 |
| Kymenlaakso | 2009 |  | 31 | 942 | 3.3 | 6.9 | 172 | 962 | 17.9 | 37.6 |
| Kymenlaakso | 2010 |  | 56 | 1,080 | 5.2 | 10.4 | 244 | 1,109 | 22.0 | 43.4 |
| Kymenlaakso | 2011 |  | 45 | 1,202 | 3.7 | 7.5 | 261 | 1,230 | 21.2 | 43.8 |
| Kymenlaakso | 2005 | BZDRs | 23 | 369 | 6.2 | 11.1 | 116 | 386 | 30.1 | 55.0 |
| Kymenlaakso | 2006 |  | 33 | 761 | 4.3 | 10.0 | 221 | 787 | 28.1 | 59.7 |
| Kymenlaakso | 2007 |  | 36 | 842 | 4.3 | 8.5 | 245 | 862 | 28.4 | 58.4 |
| Kymenlaakso | 2008 |  | 41 | 892 | 4.6 | 8.8 | 269 | 924 | 29.1 | 55.3 |
| Kymenlaakso | 2009 |  | 35 | 924 | 3.8 | 8.2 | 264 | 962 | 27.4 | 57.8 |
| Kymenlaakso | 2010 |  | 43 | 1,074 | 4.0 | 7.7 | 323 | 1,109 | 29.1 | 56.8 |
| Kymenlaakso | 2011 |  | 38 | 1,184 | 3.2 | 7.0 | 326 | 1,230 | 26.5 | 54.8 |
| 11.Länsi-Pohja | 2005 | Any | 7 | 105 | 6.7 | 14.9 | 40 | 114 | 35.1 | 68.3 |
| Länsi-Pohja | 2006 |  | 15 | 214 | 7.0 | 13.6 | 85 | 227 | 37.4 | 75.3 |
| Länsi-Pohja | 2007 |  | 20 | 213 | 9.4 | 20.9 | 99 | 232 | 42.7 | 90.2 |
| Länsi-Pohja | 2008 |  | 10 | 198 | 5.1 | 8.7 | 101 | 218 | 46.3 | 87.7 |
| Länsi-Pohja | 2009 |  | 10 | 229 | 4.4 | 7.6 | 101 | 249 | 40.6 | 77.2 |
| Länsi-Pohja | 2010 |  | 16 | 263 | 6.1 | 14.6 | 107 | 273 | 39.2 | 85.3 |
| Länsi-Pohja | 2011 |  | 11 | 230 | 4.8 | 9.3 | 107 | 261 | 41.0 | 82.3 |
| Länsi-Pohja | 2005 | Antipsychotics | 3 | 113 | 2.7 | 4.8 | 12 | 114 | 10.5 | 21.1 |
| Länsi-Pohja | 2006 |  | 10 | 227 | 4.4 | 8.7 | 25 | 227 | 11.0 | 23.2 |
| Länsi-Pohja | 2007 |  | 11 | 231 | 4.8 | 9.5 | 24 | 232 | 10.3 | 21.9 |
| Länsi-Pohja | 2008 |  | 13 | 213 | 6.1 | 11.1 | 31 | 218 | 14.2 | 26.2 |
| Länsi-Pohja | 2009 |  | 5 | 243 | 2.1 | 4.1 | 30 | 249 | 12.0 | 21.6 |
| Länsi-Pohja | 2010 |  | 8 | 271 | 3.0 | 6.6 | 32 | 273 | 11.7 | 25.0 |
| Länsi-Pohja | 2011 |  | 12 | 257 | 4.7 | 9.2 | 34 | 261 | 13.0 | 25.2 |
| Länsi-Pohja | 2005 | Antidepressants | 2 | 110 | 1.8 | 3.1 | 13 | 114 | 11.4 | 19.8 |
| Länsi-Pohja | 2006 |  | 6 | 223 | 2.7 | 5.2 | 32 | 227 | 14.1 | 28.3 |
| Länsi-Pohja | 2007 |  | 16 | 230 | 7.0 | 15.3 | 49 | 232 | 21.1 | 45.8 |
| Länsi-Pohja | 2008 |  | 6 | 215 | 2.8 | 4.1 | 44 | 218 | 20.2 | 38.3 |
| Länsi-Pohja | 2009 |  | 9 | 245 | 3.7 | 6.1 | 45 | 249 | 18.1 | 36.4 |
| Länsi-Pohja | 2010 |  | 11 | 273 | 4.0 | 9.4 | 56 | 273 | 20.5 | 43.6 |
| Länsi-Pohja | 2011 |  | 6 | 252 | 2.4 | 4.5 | 52 | 261 | 19.9 | 39.6 |
| Länsi-Pohja | 2005 | BZDRs | 6 | 107 | 5.6 | 12.7 | 30 | 114 | 26.3 | 51.5 |
| Länsi-Pohja | 2006 |  | 11 | 223 | 4.9 | 9.7 | 57 | 227 | 25.1 | 50.4 |
| Länsi-Pohja | 2007 |  | 11 | 224 | 4.9 | 11.3 | 59 | 232 | 25.4 | 54.7 |
| Länsi-Pohja | 2008 |  | 7 | 212 | 3.3 | 5.3 | 63 | 218 | 28.9 | 55.7 |
| Länsi-Pohja | 2009 |  | 5 | 242 | 2.1 | 3.5 | 60 | 249 | 24.1 | 46.5 |
| Länsi-Pohja | 2010 |  | 7 | 269 | 2.6 | 6.3 | 53 | 273 | 19.4 | 43.4 |
| Länsi-Pohja | 2011 |  | 9 | 252 | 3.6 | 6.4 | 62 | 261 | 23.8 | 47.7 |
| 12.Lappi | 2005 | Any | 15 | 178 | 8.4 | 13.9 | 89 | 200 | 44.5 | 89.0 |
| Lappi | 2006 |  | 30 | 356 | 8.4 | 16.8 | 182 | 388 | 46.9 | 95.2 |
| Lappi | 2007 |  | 35 | 398 | 8.8 | 18.9 | 216 | 436 | 49.5 | 105.9 |
| Lappi | 2008 |  | 29 | 434 | 6.7 | 14.4 | 222 | 493 | 45.0 | 92.2 |
| Lappi | 2009 |  | 29 | 432 | 6.7 | 12.7 | 217 | 493 | 44.0 | 86.3 |
| Lappi | 2010 |  | 30 | 418 | 7.2 | 15.3 | 190 | 452 | 42.0 | 86.3 |
| Lappi | 2011 |  | 32 | 379 | 8.4 | 16.7 | 189 | 424 | 44.6 | 85.2 |
| Lappi | 2005 | Antipsychotics | 17 | 197 | 8.6 | 16.0 | 32 | 200 | 16.0 | 35.1 |
| Lappi | 2006 |  | 21 | 384 | 5.5 | 10.7 | 64 | 388 | 16.5 | 34.4 |
| Lappi | 2007 |  | 28 | 430 | 6.5 | 15.4 | 78 | 436 | 17.9 | 39.3 |
| Lappi | 2008 |  | 33 | 478 | 6.9 | 16.6 | 89 | 493 | 18.1 | 38.6 |
| Lappi | 2009 |  | 23 | 487 | 4.7 | 10.0 | 75 | 493 | 15.2 | 30.1 |
| Lappi | 2010 |  | 20 | 446 | 4.5 | 9.5 | 61 | 452 | 13.5 | 28.0 |
| Lappi | 2011 |  | 30 | 416 | 7.2 | 13.6 | 65 | 424 | 15.3 | 31.1 |
| Lappi | 2005 | Antidepressants | 7 | 193 | 3.6 | 4.8 | 47 | 200 | 23.5 | 44.9 |
| Lappi | 2006 |  | 9 | 379 | 2.4 | 4.9 | 84 | 388 | 21.6 | 43.8 |
| Lappi | 2007 |  | 18 | 432 | 4.2 | 6.9 | 88 | 436 | 20.2 | 41.9 |
| Lappi | 2008 |  | 17 | 478 | 3.6 | 7.0 | 93 | 493 | 18.9 | 36.7 |
| Lappi | 2009 |  | 15 | 478 | 3.1 | 6.5 | 84 | 493 | 17.0 | 32.1 |
| Lappi | 2010 |  | 13 | 443 | 2.9 | 5.7 | 75 | 452 | 16.6 | 33.4 |
| Lappi | 2011 |  | 17 | 411 | 4.1 | 8.1 | 89 | 424 | 21.0 | 40.7 |
| Lappi | 2005 | BZDRs | 11 | 195 | 5.6 | 11.2 | 43 | 200 | 21.5 | 46.9 |
| Lappi | 2006 |  | 21 | 376 | 5.6 | 9.8 | 105 | 388 | 27.1 | 54.8 |
| Lappi | 2007 |  | 30 | 423 | 7.1 | 15.1 | 137 | 436 | 31.4 | 67.0 |
| Lappi | 2008 |  | 18 | 475 | 3.8 | 7.7 | 134 | 493 | 27.2 | 57.2 |
| Lappi | 2009 |  | 26 | 464 | 5.6 | 10.4 | 144 | 493 | 29.2 | 58.8 |
| Lappi | 2010 |  | 14 | 432 | 3.2 | 6.9 | 119 | 452 | 26.3 | 54.7 |
| Lappi | 2011 |  | 19 | 405 | 4.7 | 9.2 | 108 | 424 | 25.5 | 50.0 |
| 13.Päijät-Häme | 2005 | Any | 28 | 314 | 8.9 | 18.2 | 168 | 356 | 47.2 | 88.2 |
| Päijät-Häme | 2006 |  | 48 | 646 | 7.4 | 14.8 | 353 | 729 | 48.4 | 96.0 |
| Päijät-Häme | 2007 |  | 57 | 689 | 8.3 | 18.3 | 377 | 775 | 48.6 | 95.5 |
| Päijät-Häme | 2008 |  | 77 | 799 | 9.6 | 19.6 | 445 | 896 | 49.7 | 96.6 |
| Päijät-Häme | 2009 |  | 68 | 808 | 8.4 | 17.5 | 417 | 896 | 46.5 | 93.5 |
| Päijät-Häme | 2010 |  | 67 | 785 | 8.5 | 17.7 | 385 | 864 | 44.6 | 91.5 |
| Päijät-Häme | 2011 |  | 85 | 883 | 9.6 | 18.5 | 488 | 1,008 | 48.4 | 89.5 |
| Päijät-Häme | 2005 | Antipsychotics | 20 | 349 | 5.7 | 10.6 | 61 | 356 | 17.1 | 32.2 |
| Päijät-Häme | 2006 |  | 37 | 718 | 5.2 | 10.2 | 119 | 729 | 16.3 | 32.3 |
| Päijät-Häme | 2007 |  | 37 | 764 | 4.8 | 10.0 | 132 | 775 | 17.0 | 33.5 |
| Päijät-Häme | 2008 |  | 56 | 883 | 6.3 | 12.5 | 143 | 896 | 16.0 | 30.8 |
| Päijät-Häme | 2009 |  | 43 | 886 | 4.9 | 10.1 | 135 | 896 | 15.1 | 30.4 |
| Päijät-Häme | 2010 |  | 51 | 849 | 6.0 | 12.4 | 132 | 864 | 15.3 | 31.9 |
| Päijät-Häme | 2011 |  | 71 | 998 | 7.1 | 13.5 | 185 | 1,008 | 18.4 | 34.5 |
| Päijät-Häme | 2005 | Antidepressants | 20 | 347 | 5.8 | 11.0 | 73 | 356 | 20.5 | 37.0 |
| Päijät-Häme | 2006 |  | 41 | 707 | 5.8 | 11.0 | 163 | 729 | 22.4 | 44.1 |
| Päijät-Häme | 2007 |  | 35 | 757 | 4.6 | 9.0 | 166 | 775 | 21.4 | 42.2 |
| Päijät-Häme | 2008 |  | 40 | 869 | 4.6 | 9.0 | 213 | 896 | 23.8 | 45.7 |
| Päijät-Häme | 2009 |  | 47 | 876 | 5.4 | 10.6 | 209 | 896 | 23.3 | 46.7 |
| Päijät-Häme | 2010 |  | 41 | 848 | 4.8 | 9.8 | 192 | 864 | 22.2 | 46.2 |
| Päijät-Häme | 2011 |  | 50 | 969 | 5.2 | 9.4 | 251 | 1,008 | 24.9 | 46.1 |
| Päijät-Häme | 2005 | BZDRs | 23 | 338 | 6.8 | 12.4 | 114 | 356 | 32.0 | 58.8 |
| Päijät-Häme | 2006 |  | 35 | 698 | 5.0 | 9.7 | 231 | 729 | 31.7 | 63.3 |
| Päijät-Häme | 2007 |  | 51 | 733 | 7.0 | 14.2 | 250 | 775 | 32.3 | 63.2 |
| Päijät-Häme | 2008 |  | 49 | 858 | 5.7 | 11.6 | 268 | 896 | 29.9 | 58.7 |
| Päijät-Häme | 2009 |  | 42 | 860 | 4.9 | 10.1 | 239 | 896 | 26.7 | 54.3 |
| Päijät-Häme | 2010 |  | 38 | 838 | 4.5 | 9.3 | 218 | 864 | 25.2 | 51.4 |
| Päijät-Häme | 2011 |  | 59 | 969 | 6.1 | 11.6 | 278 | 1,008 | 27.6 | 50.4 |
| 14.Pirkanmaa | 2005 | Any | 68 | 719 | 9.5 | 17.2 | 393 | 816 | 48.2 | 86.7 |
| Pirkanmaa | 2006 |  | 124 | 1,367 | 9.1 | 18.5 | 755 | 1,518 | 49.7 | 99.5 |
| Pirkanmaa | 2007 |  | 121 | 1,422 | 8.5 | 17.3 | 832 | 1,606 | 51.8 | 102.2 |
| Pirkanmaa | 2008 |  | 110 | 1,537 | 7.2 | 14.7 | 876 | 1,719 | 51.0 | 101.6 |
| Pirkanmaa | 2009 |  | 129 | 1539 | 8.4 | 17.2 | 860 | 1,710 | 50.3 | 99.3 |
| Pirkanmaa | 2010 |  | 149 | 1,634 | 9.1 | 18.9 | 936 | 1,837 | 51.0 | 102.0 |
| Pirkanmaa | 2011 |  | 142 | 1,799 | 7.9 | 15.5 | 992 | 2,002 | 49.6 | 97.6 |
| Pirkanmaa | 2005 | Antipsychotics | 60 | 802 | 7.5 | 13.8 | 114 | 816 | 14.0 | 25.5 |
| Pirkanmaa | 2006 |  | 82 | 1,496 | 5.5 | 11.1 | 228 | 1,518 | 15.0 | 30.0 |
| Pirkanmaa | 2007 |  | 101 | 1,592 | 6.3 | 12.5 | 246 | 1,606 | 15.3 | 30.1 |
| Pirkanmaa | 2008 |  | 93 | 1,703 | 5.5 | 11.0 | 261 | 1,719 | 15.2 | 30.1 |
| Pirkanmaa | 2009 |  | 106 | 1,688 | 6.3 | 12.2 | 273 | 1,710 | 16.0 | 31.2 |
| Pirkanmaa | 2010 |  | 129 | 1,809 | 7.1 | 14.4 | 314 | 1,837 | 17.1 | 34.0 |
| Pirkanmaa | 2011 |  | 136 | 1,976 | 6.9 | 13.6 | 357 | 2,002 | 17.8 | 35.4 |
| Pirkanmaa | 2005 | Antidepressants | 40 | 783 | 5.1 | 8.9 | 207 | 816 | 25.4 | 44.8 |
| Pirkanmaa | 2006 |  | 84 | 1,472 | 5.7 | 11.6 | 379 | 1,518 | 25.0 | 49.4 |
| Pirkanmaa | 2007 |  | 65 | 1,550 | 4.2 | 8.3 | 424 | 1,606 | 26.4 | 51.4 |
| Pirkanmaa | 2008 |  | 88 | 1,663 | 5.3 | 10.5 | 484 | 1,719 | 28.2 | 55.8 |
| Pirkanmaa | 2009 |  | 88 | 1,662 | 5.3 | 10.6 | 476 | 1,710 | 27.8 | 55.2 |
| Pirkanmaa | 2010 |  | 81 | 1,767 | 4.6 | 9.3 | 492 | 1,837 | 26.8 | 53.8 |
| Pirkanmaa | 2011 |  | 92 | 1,947 | 4.7 | 9.3 | 535 | 2,002 | 26.7 | 53.1 |
| Pirkanmaa | 2005 | BZDRs | 45 | 787 | 5.7 | 10.4 | 245 | 816 | 30.0 | 54.7 |
| Pirkanmaa | 2006 |  | 66 | 1,468 | 4.5 | 9.1 | 461 | 1,518 | 30.4 | 61.2 |
| Pirkanmaa | 2007 |  | 65 | 1,525 | 4.3 | 8.7 | 496 | 1,606 | 30.9 | 61.4 |
| Pirkanmaa | 2008 |  | 58 | 1,656 | 3.5 | 7.0 | 491 | 1,719 | 28.6 | 57.2 |
| Pirkanmaa | 2009 |  | 64 | 1,651 | 3.9 | 7.7 | 456 | 1,710 | 26.7 | 52.5 |
| Pirkanmaa | 2010 |  | 84 | 1,775 | 4.7 | 9.7 | 496 | 1,837 | 27.0 | 54.2 |
| Pirkanmaa | 2011 |  | 83 | 1,935 | 4.3 | 8.5 | 503 | 2,002 | 25.1 | 49.4 |
| 15.Pohjois-Karjala | 2005 | Any | 22 | 257 | 8.6 | 17.2 | 129 | 291 | 44.3 | 81.1 |
| Pohjois-Karjala | 2006 |  | 52 | 587 | 8.9 | 17.6 | 293 | 650 | 45.1 | 89.7 |
| Pohjois-Karjala | 2007 |  | 48 | 645 | 7.4 | 15.7 | 332 | 721 | 46.0 | 89.9 |
| Pohjois-Karjala | 2008 |  | 57 | 719 | 7.9 | 16.8 | 356 | 801 | 44.4 | 91.9 |
| Pohjois-Karjala | 2009 |  | 55 | 688 | 8.0 | 16.7 | 357 | 759 | 47.0 | 97.6 |
| Pohjois-Karjala | 2010 |  | 42 | 573 | 7.3 | 15.2 | 308 | 650 | 47.4 | 94.4 |
| Pohjois-Karjala | 2011 |  | 55 | 680 | 8.1 | 16.9 | 344 | 742 | 46.4 | 94.6 |
| Pohjois-Karjala | 2005 | Antipsychotics | 21 | 282 | 7.4 | 14.1 | 56 | 291 | 19.2 | 35.6 |
| Pohjois-Karjala | 2006 |  | 32 | 638 | 5.0 | 9.7 | 110 | 650 | 16.9 | 34.5 |
| Pohjois-Karjala | 2007 |  | 43 | 707 | 6.1 | 12.7 | 117 | 721 | 16.2 | 32.5 |
| Pohjois-Karjala | 2008 |  | 49 | 784 | 6.3 | 12.9 | 136 | 801 | 17.0 | 35.1 |
| Pohjois-Karjala | 2009 |  | 45 | 749 | 6.0 | 12.4 | 138 | 759 | 18.2 | 38.2 |
| Pohjois-Karjala | 2010 |  | 43 | 632 | 6.8 | 13.2 | 124 | 650 | 19.1 | 38.2 |
| Pohjois-Karjala | 2011 |  | 48 | 729 | 6.6 | 13.1 | 141 | 742 | 19.0 | 37.9 |
| Pohjois-Karjala | 2005 | Antidepressants | 11 | 283 | 3.9 | 7.7 | 61 | 291 | 21.0 | 38.8 |
| Pohjois-Karjala | 2006 |  | 36 | 632 | 5.7 | 10.9 | 148 | 650 | 22.8 | 44.0 |
| Pohjois-Karjala | 2007 |  | 31 | 699 | 4.4 | 8.3 | 166 | 721 | 23.0 | 43.9 |
| Pohjois-Karjala | 2008 |  | 35 | 784 | 4.5 | 9.3 | 180 | 801 | 22.5 | 46.6 |
| Pohjois-Karjala | 2009 |  | 37 | 744 | 5.0 | 10.3 | 175 | 759 | 23.1 | 47.7 |
| Pohjois-Karjala | 2010 |  | 34 | 636 | 5.3 | 10.5 | 146 | 650 | 22.5 | 44.5 |
| Pohjois-Karjala | 2011 |  | 32 | 728 | 4.4 | 9.2 | 184 | 742 | 24.8 | 51.5 |
| Pohjois-Karjala | 2005 | BZDRs | 14 | 282 | 5.0 | 8.6 | 63 | 291 | 21.6 | 38.2 |
| Pohjois-Karjala | 2006 |  | 41 | 632 | 6.5 | 12.7 | 152 | 650 | 23.4 | 46.8 |
| Pohjois-Karjala | 2007 |  | 42 | 696 | 6.0 | 12.3 | 203 | 721 | 28.2 | 55.0 |
| Pohjois-Karjala | 2008 |  | 28 | 770 | 3.6 | 7.4 | 194 | 801 | 24.2 | 50.2 |
| Pohjois-Karjala | 2009 |  | 26 | 732 | 3.6 | 7.4 | 187 | 759 | 24.6 | 51.8 |
| Pohjois-Karjala | 2010 |  | 22 | 624 | 3.5 | 7.1 | 146 | 650 | 22.5 | 44.4 |
| Pohjois-Karjala | 2011 |  | 24 | 718 | 3.3 | 6.8 | 151 | 742 | 20.4 | 41.9 |
| 16.Pohjois-Pohjanmaa | 2005 | Any | 62 | 642 | 9.7 | 17.8 | 351 | 731 | 48.0 | 81.7 |
| Pohjois-Pohjanmaa | 2006 |  | 87 | 1,244 | 7.0 | 14.6 | 659 | 1,392 | 47.3 | 96.9 |
| Pohjois-Pohjanmaa | 2007 |  | 80 | 1,122 | 7.1 | 14.7 | 567 | 1,244 | 45.6 | 90.8 |
| Pohjois-Pohjanmaa | 2008 |  | 93 | 1,153 | 8.1 | 17.5 | 617 | 1,270 | 48.6 | 102.2 |
| Pohjois-Pohjanmaa | 2009 |  | 91 | 1,302 | 7.0 | 14.2 | 743 | 1,460 | 50.9 | 102.0 |
| Pohjois-Pohjanmaa | 2010 |  | 90 | 1,376 | 6.5 | 12.9 | 771 | 1,524 | 50.6 | 98.2 |
| Pohjois-Pohjanmaa | 2011 |  | 94 | 1,444 | 6.5 | 13.7 | 754 | 1,593 | 47.3 | 98.8 |
| Pohjois-Pohjanmaa | 2005 | Antipsychotics | 38 | 717 | 5.3 | 9.7 | 94 | 731 | 12.9 | 22.2 |
| Pohjois-Pohjanmaa | 2006 |  | 58 | 1,374 | 4.2 | 8.6 | 182 | 1,392 | 13.1 | 26.7 |
| Pohjois-Pohjanmaa | 2007 |  | 55 | 1,230 | 4.5 | 8.8 | 164 | 1,244 | 13.2 | 25.7 |
| Pohjois-Pohjanmaa | 2008 |  | 71 | 1,247 | 5.7 | 12.1 | 197 | 1,270 | 15.5 | 32.1 |
| Pohjois-Pohjanmaa | 2009 |  | 87 | 1,435 | 6.1 | 12.3 | 251 | 1,460 | 17.2 | 34.0 |
| Pohjois-Pohjanmaa | 2010 |  | 70 | 1,495 | 4.7 | 9.0 | 246 | 1,524 | 16.1 | 31.1 |
| Pohjois-Pohjanmaa | 2011 |  | 90 | 1,558 | 5.8 | 12.0 | 271 | 1,593 | 17.0 | 35.6 |
| Pohjois-Pohjanmaa | 2005 | Antidepressants | 43 | 702 | 6.1 | 10.7 | 195 | 731 | 26.7 | 45.0 |
| Pohjois-Pohjanmaa | 2006 |  | 58 | 1,362 | 4.3 | 8.9 | 350 | 1,392 | 25.1 | 51.2 |
| Pohjois-Pohjanmaa | 2007 |  | 59 | 1,201 | 4.9 | 9.8 | 293 | 1,244 | 23.6 | 46.2 |
| Pohjois-Pohjanmaa | 2008 |  | 59 | 1,238 | 4.8 | 10.2 | 309 | 1,270 | 24.3 | 50.6 |
| Pohjois-Pohjanmaa | 2009 |  | 61 | 1,428 | 4.3 | 8.5 | 363 | 1,460 | 24.9 | 49.7 |
| Pohjois-Pohjanmaa | 2010 |  | 60 | 1,479 | 4.1 | 8.2 | 397 | 1,524 | 26.0 | 50.6 |
| Pohjois-Pohjanmaa | 2011 |  | 52 | 1,558 | 3.3 | 6.9 | 391 | 1,593 | 24.5 | 51.7 |
| Pohjois-Pohjanmaa | 2005 | BZDRs | 50 | 700 | 7.1 | 12.0 | 233 | 731 | 31.9 | 53.9 |
| Pohjois-Pohjanmaa | 2006 |  | 65 | 1,324 | 4.9 | 10.0 | 414 | 1,392 | 29.7 | 61.6 |
| Pohjois-Pohjanmaa | 2007 |  | 40 | 1,195 | 3.3 | 6.9 | 345 | 1,244 | 27.7 | 55.7 |
| Pohjois-Pohjanmaa | 2008 |  | 51 | 1,227 | 4.2 | 9.0 | 356 | 1,270 | 28.0 | 59.2 |
| Pohjois-Pohjanmaa | 2009 |  | 54 | 1,408 | 3.8 | 7.7 | 429 | 1,460 | 29.4 | 59.3 |
| Pohjois-Pohjanmaa | 2010 |  | 51 | 1,468 | 3.5 | 6.7 | 420 | 1,524 | 27.6 | 53.7 |
| Pohjois-Pohjanmaa | 2011 |  | 46 | 1,541 | 3.0 | 6.2 | 381 | 1,593 | 23.9 | 49.7 |
| 17.Pohjois-Savo | 2005 | Any | 47 | 403 | 11.7 | 24.5 | 210 | 451 | 46.6 | 94.1 |
| Pohjois-Savo | 2006 |  | 100 | 829 | 12.1 | 24.5 | 477 | 931 | 51.2 | 102.6 |
| Pohjois-Savo | 2007 |  | 74 | 905 | 8.2 | 16.7 | 532 | 1,040 | 51.2 | 101.4 |
| Pohjois-Savo | 2008 |  | 86 | 1,066 | 8.1 | 15.7 | 576 | 1,174 | 49.1 | 93.5 |
| Pohjois-Savo | 2009 |  | 94 | 1,134 | 8.3 | 18.5 | 626 | 1,269 | 49.3 | 105.1 |
| Pohjois-Savo | 2010 |  | 100 | 1,131 | 8.8 | 17.3 | 605 | 1,268 | 47.7 | 92.1 |
| Pohjois-Savo | 2011 |  | 104 | 1,265 | 8.2 | 16.8 | 635 | 1,404 | 45.2 | 88.9 |
| Pohjois-Savo | 2005 | Antipsychotics | 31 | 441 | 7.0 | 13.8 | 81 | 451 | 18.0 | 35.7 |
| Pohjois-Savo | 2006 |  | 61 | 916 | 6.7 | 13.8 | 176 | 931 | 18.9 | 38.3 |
| Pohjois-Savo | 2007 |  | 69 | 1,022 | 6.8 | 13.3 | 203 | 1,040 | 19.5 | 38.5 |
| Pohjois-Savo | 2008 |  | 72 | 1,156 | 6.2 | 11.8 | 223 | 1,174 | 19.0 | 36.1 |
| Pohjois-Savo | 2009 |  | 83 | 1,243 | 6.7 | 14.4 | 267 | 1,269 | 21.0 | 45.0 |
| Pohjois-Savo | 2010 |  | 91 | 1,247 | 7.3 | 14.1 | 273 | 1,268 | 21.5 | 41.9 |
| Pohjois-Savo | 2011 |  | 106 | 1,377 | 7.7 | 15.2 | 269 | 1,404 | 19.2 | 38.0 |
| Pohjois-Savo | 2005 | Antidepressants | 33 | 433 | 7.6 | 16.6 | 113 | 451 | 25.1 | 51.1 |
| Pohjois-Savo | 2006 |  | 65 | 900 | 7.2 | 14.2 | 247 | 931 | 26.5 | 52.7 |
| Pohjois-Savo | 2007 |  | 62 | 1,001 | 6.2 | 12.2 | 296 | 1,040 | 28.5 | 56.4 |
| Pohjois-Savo | 2008 |  | 49 | 1,146 | 4.3 | 8.3 | 302 | 1,174 | 25.7 | 49.1 |
| Pohjois-Savo | 2009 |  | 57 | 1,242 | 4.6 | 9.9 | 319 | 1,269 | 25.1 | 53.5 |
| Pohjois-Savo | 2010 |  | 58 | 1,230 | 4.7 | 9.0 | 309 | 1,268 | 24.4 | 47.0 |
| Pohjois-Savo | 2011 |  | 64 | 1,370 | 4.7 | 9.2 | 327 | 1,404 | 23.3 | 46.1 |
| Pohjois-Savo | 2005 | BZDRs | 25 | 435 | 5.7 | 11.6 | 121 | 451 | 26.8 | 55.0 |
| Pohjois-Savo | 2006 |  | 73 | 902 | 8.1 | 15.9 | 273 | 931 | 29.3 | 58.7 |
| Pohjois-Savo | 2007 |  | 46 | 994 | 4.6 | 9.1 | 269 | 1,040 | 25.9 | 51.3 |
| Pohjois-Savo | 2008 |  | 57 | 1,145 | 5.0 | 9.5 | 288 | 1,174 | 24.5 | 46.9 |
| Pohjois-Savo | 2009 |  | 59 | 1,222 | 4.8 | 10.3 | 318 | 1,269 | 25.1 | 53.4 |
| Pohjois-Savo | 2010 |  | 50 | 1,217 | 4.1 | 7.9 | 288 | 1,268 | 22.7 | 43.5 |
| Pohjois-Savo | 2011 |  | 48 | 1,352 | 3.6 | 7.3 | 302 | 1,404 | 21.5 | 41.9 |
| 18.Satakunta | 2005 | Any | 35 | 328 | 10.7 | 22.3 | 178 | 372 | 47.8 | 91.0 |
| Satakunta | 2006 |  | 45 | 654 | 6.9 | 13.6 | 336 | 735 | 45.7 | 88.7 |
| Satakunta | 2007 |  | 63 | 686 | 9.2 | 18.6 | 377 | 773 | 48.8 | 96.1 |
| Satakunta | 2008 |  | 65 | 745 | 8.7 | 18.4 | 412 | 823 | 50.1 | 101.6 |
| Satakunta | 2009 |  | 58 | 751 | 7.7 | 15.5 | 389 | 848 | 45.9 | 93.1 |
| Satakunta | 2010 |  | 54 | 740 | 7.3 | 15.2 | 360 | 800 | 45.0 | 90.0 |
| Satakunta | 2011 |  | 64 | 772 | 8.3 | 16.8 | 378 | 838 | 45.1 | 89.0 |
| Satakunta | 2005 | Antipsychotics | 31 | 363 | 8.5 | 16.5 | 59 | 372 | 15.9 | 30.2 |
| Satakunta | 2006 |  | 35 | 713 | 4.9 | 9.6 | 113 | 735 | 15.4 | 30.0 |
| Satakunta | 2007 |  | 58 | 763 | 7.6 | 14.7 | 127 | 773 | 16.4 | 33.0 |
| Satakunta | 2008 |  | 63 | 810 | 7.8 | 15.8 | 154 | 823 | 18.7 | 37.9 |
| Satakunta | 2009 |  | 62 | 831 | 7.5 | 14.8 | 163 | 848 | 19.2 | 40.1 |
| Satakunta | 2010 |  | 46 | 791 | 5.8 | 11.8 | 139 | 800 | 17.4 | 35.1 |
| Satakunta | 2011 |  | 48 | 825 | 5.8 | 11.3 | 152 | 838 | 18.1 | 36.3 |
| Satakunta | 2005 | Antidepressants | 17 | 359 | 4.7 | 9.0 | 110 | 372 | 29.6 | 55.0 |
| Satakunta | 2006 |  | 27 | 717 | 3.8 | 7.4 | 188 | 735 | 25.6 | 49.3 |
| Satakunta | 2007 |  | 47 | 743 | 6.3 | 12.4 | 211 | 773 | 27.3 | 53.4 |
| Satakunta | 2008 |  | 39 | 793 | 4.9 | 9.8 | 231 | 823 | 28.1 | 57.2 |
| Satakunta | 2009 |  | 33 | 821 | 4.0 | 8.0 | 217 | 848 | 25.6 | 51.3 |
| Satakunta | 2010 |  | 31 | 785 | 3.9 | 8.2 | 191 | 800 | 23.9 | 48.0 |
| Satakunta | 2011 |  | 37 | 814 | 4.5 | 9.0 | 214 | 838 | 25.5 | 51.0 |
| Satakunta | 2005 | BZDRs | 18 | 353 | 5.1 | 9.9 | 93 | 372 | 25.0 | 47.1 |
| Satakunta | 2006 |  | 33 | 708 | 4.7 | 8.9 | 204 | 735 | 27.8 | 53.6 |
| Satakunta | 2007 |  | 34 | 748 | 4.5 | 9.2 | 225 | 773 | 29.1 | 56.8 |
| Satakunta | 2008 |  | 24 | 804 | 3.0 | 6.2 | 181 | 823 | 22.0 | 44.5 |
| Satakunta | 2009 |  | 31 | 823 | 3.8 | 9.8 | 156 | 848 | 18.4 | 38.3 |
| Satakunta | 2010 |  | 20 | 777 | 2.6 | 5.4 | 147 | 800 | 18.4 | 36.1 |
| Satakunta | 2011 |  | 27 | 819 | 3.3 | 6.9 | 144 | 838 | 17.2 | 33.8 |
| 19.Vaasa | 2005 | Any | 20 | 203 | 9.9 | 15.8 | 119 | 231 | 51.5 | 88.1 |
| Vaasa | 2006 |  | 42 | 449 | 9.4 | 18.8 | 263 | 496 | 53.0 | 115.5 |
| Vaasa | 2007 |  | 37 | 465 | 8.0 | 16.5 | 273 | 523 | 52.2 | 103.0 |
| Vaasa | 2008 |  | 38 | 495 | 7.7 | 15.2 | 281 | 547 | 51.4 | 100.9 |
| Vaasa | 2009 |  | 34 | 524 | 6.5 | 13.3 | 276 | 579 | 47.7 | 94.8 |
| Vaasa | 2010 |  | 40 | 534 | 7.5 | 15.5 | 274 | 598 | 45.8 | 91.1 |
| Vaasa | 2011 |  | 40 | 557 | 7.2 | 15.0 | 318 | 623 | 51.0 | 103.1 |
| Vaasa | 2005 | Antipsychotics | 16 | 226 | 7.1 | 12.6 | 44 | 231 | 19.0 | 34.9 |
| Vaasa | 2006 |  | 23 | 484 | 4.8 | 10.4 | 85 | 496 | 17.1 | 39.4 |
| Vaasa | 2007 |  | 28 | 512 | 5.5 | 11.2 | 88 | 523 | 16.8 | 33.4 |
| Vaasa | 2008 |  | 29 | 536 | 5.4 | 10.9 | 93 | 547 | 17.0 | 33.8 |
| Vaasa | 2009 |  | 27 | 569 | 4.7 | 9.6 | 86 | 579 | 14.9 | 29.5 |
| Vaasa | 2010 |  | 31 | 588 | 5.3 | 10.4 | 93 | 598 | 15.6 | 30.4 |
| Vaasa | 2011 |  | 27 | 607 | 4.4 | 9.1 | 112 | 623 | 18.0 | 35.9 |
| Vaasa | 2005 | Antidepressants | 19 | 225 | 8.4 | 12.2 | 71 | 231 | 30.7 | 51.5 |
| Vaasa | 2006 |  | 27 | 476 | 5.7 | 11.5 | 156 | 496 | 31.5 | 67.7 |
| Vaasa | 2007 |  | 20 | 505 | 4.0 | 8.0 | 161 | 523 | 30.8 | 60.8 |
| Vaasa | 2008 |  | 19 | 534 | 3.6 | 7.1 | 156 | 547 | 28.5 | 55.3 |
| Vaasa | 2009 |  | 19 | 566 | 3.4 | 6.5 | 155 | 579 | 26.8 | 53.0 |
| Vaasa | 2010 |  | 20 | 578 | 3.5 | 6.9 | 148 | 598 | 24.7 | 49.2 |
| Vaasa | 2011 |  | 29 | 608 | 4.8 | 9.8 | 173 | 623 | 27.8 | 55.5 |
| Vaasa | 2005 | BZDRs | 11 | 222 | 5.0 | 8.1 | 56 | 231 | 24.2 | 41.1 |
| Vaasa | 2006 |  | 18 | 486 | 3.7 | 7.9 | 128 | 496 | 25.8 | 55.0 |
| Vaasa | 2007 |  | 27 | 506 | 5.3 | 10.5 | 151 | 523 | 28.9 | 56.8 |
| Vaasa | 2008 |  | 26 | 530 | 4.9 | 9.9 | 149 | 547 | 27.2 | 54.4 |
| Vaasa | 2009 |  | 25 | 564 | 4.4 | 9.2 | 146 | 579 | 25.2 | 50.7 |
| Vaasa | 2010 |  | 23 | 576 | 4.0 | 8.0 | 143 | 598 | 23.9 | 47.8 |
| Vaasa | 2011 |  | 22 | 594 | 3.7 | 8.0 | 166 | 623 | 26.6 | 54.4 |
| 20.Varsinais-Suomi | 2005 | Any | 64 | 710 | 9.0 | 16.8 | 393 | 794 | 49.5 | 89.4 |
| Varsinais-Suomi | 2006 |  | 103 | 1,406 | 7.3 | 15.7 | 769 | 1,573 | 48.9 | 100.5 |
| Varsinais-Suomi | 2007 |  | 105 | 1,336 | 7.9 | 15.0 | 726 | 1,479 | 49.1 | 91.8 |
| Varsinais-Suomi | 2008 |  | 102 | 1,323 | 7.7 | 15.7 | 719 | 1,465 | 49.1 | 97.8 |
| Varsinais-Suomi | 2009 |  | 104 | 1,451 | 7.2 | 14.8 | 775 | 1,598 | 48.5 | 98.5 |
| Varsinais-Suomi | 2010 |  | 125 | 1,431 | 8.7 | 17.5 | 749 | 1,565 | 47.9 | 94.0 |
| Varsinais-Suomi | 2011 |  | 132 | 1,544 | 8.5 | 17.6 | 816 | 1,698 | 48.1 | 94.6 |
| Varsinais-Suomi | 2005 | Antipsychotics | 63 | 784 | 8.0 | 14.6 | 121 | 794 | 15.2 | 28.1 |
| Varsinais-Suomi | 2006 |  | 87 | 1,552 | 5.6 | 11.5 | 255 | 1,573 | 16.2 | 33.5 |
| Varsinais-Suomi | 2007 |  | 82 | 1,460 | 5.6 | 10.6 | 237 | 1,479 | 16.0 | 29.9 |
| Varsinais-Suomi | 2008 |  | 102 | 1,441 | 7.1 | 14.0 | 270 | 1,465 | 18.4 | 36.7 |
| Varsinais-Suomi | 2009 |  | 84 | 1,578 | 5.3 | 10.7 | 264 | 1,598 | 16.5 | 33.5 |
| Varsinais-Suomi | 2010 |  | 114 | 1,540 | 7.4 | 14.5 | 271 | 1,565 | 17.3 | 34.0 |
| Varsinais-Suomi | 2011 |  | 94 | 1,667 | 5.6 | 11.4 | 290 | 1,698 | 17.1 | 33.7 |
| Varsinais-Suomi | 2005 | Antidepressants | 36 | 773 | 4.7 | 8.3 | 187 | 794 | 23.6 | 43.1 |
| Varsinais-Suomi | 2006 |  | 62 | 1,537 | 4.0 | 8.2 | 356 | 1,573 | 22.6 | 46.2 |
| Varsinais-Suomi | 2007 |  | 65 | 1,446 | 4.5 | 8.6 | 330 | 1,479 | 22.3 | 42.2 |
| Varsinais-Suomi | 2008 |  | 55 | 1,438 | 3.8 | 7.7 | 326 | 1,465 | 22.3 | 44.6 |
| Varsinais-Suomi | 2009 |  | 54 | 1,560 | 3.5 | 7.1 | 345 | 1,598 | 21.6 | 44.1 |
| Varsinais-Suomi | 2010 |  | 57 | 1,531 | 3.7 | 7.3 | 346 | 1,565 | 22.1 | 43.9 |
| Varsinais-Suomi | 2011 |  | 83 | 1,672 | 5.0 | 10.1 | 381 | 1,698 | 22.4 | 44.8 |
| Varsinais-Suomi | 2005 | BZDRs | 45 | 765 | 5.9 | 10.5 | 250 | 794 | 31.5 | 56.8 |
| Varsinais-Suomi | 2006 |  | 71 | 1,503 | 4.7 | 10.1 | 497 | 1,573 | 31.6 | 65.0 |
| Varsinais-Suomi | 2007 |  | 57 | 1,426 | 4.0 | 7.4 | 455 | 1,479 | 30.8 | 56.8 |
| Varsinais-Suomi | 2008 |  | 73 | 1,411 | 5.2 | 10.3 | 446 | 1,465 | 30.4 | 60.3 |
| Varsinais-Suomi | 2009 |  | 66 | 1,545 | 4.3 | 8.5 | 470 | 1,598 | 29.4 | 59.3 |
| Varsinais-Suomi | 2010 |  | 65 | 1,508 | 4.3 | 8.6 | 426 | 1,565 | 27.2 | 53.4 |
| Varsinais-Suomi | 2011 |  | 83 | 1,643 | 5.1 | 10.0 | 460 | 1,698 | 27.1 | 52.7 |
